# Phagocytosis of microbial symbionts supports embryonic nutrition in the sponge *Halichondria panicea*

**DOI:** 10.1186/s40168-026-02473-y

**Published:** 2026-08-26

**Authors:** Marta Turon, Cristina Díez-Vives, Tyler J. Carrier, Laura de la Cruz-Castillejo, Maria Conejero, Leon Steiner, Sabrina Jung, Lucía Pita, Vasiliki Koutsouveli, Sören Franzenburg, Ute Hentschel, Ana Riesgo

**Affiliations:** 1 Department of Biodiversity and Evolutionary Biology, Museo Nacional de Ciencias Naturales (MNCN-CSIC), c/José Gutiérrez Abascal 2, Madrid, 28006 Spain; 2 Marine Ecology Department, Centre d’Estudis Avançats de Blanes (CEAB-CSIC), C/Accés Cala Sant Francesc 14, Blanes, 17300 Spain; 3 Department of Systems Biology, Centro Nacional de Biotecnología, C/Darwin, 3, Madrid, 28049 Spain; 4 Centre for Marine Science and Innovation and School of Biological, Earth and Environmental Sciences, The University of New South Wales, Sydney, NSW Australia; 5 Research Unit Marine Symbioses, GEOMAR Helmholtz Centre for Ocean Research, Wischhofstr. 1-3, Kiel, 24148 Germany; 6 Zoological Institute, Christian-Albrechts University of Kiel, Am Botanischen Garten 1-9, Kiel, 24118 Germany; 7 Department of Marine Integrated Ecology, Institute de Investigaciones Marinas (IIM-CSIC), R/Eduardo Cabello 6, Vigo, 36208 Spain; 8 Institute of Clinical Molecular Biology, Christian-Albrechts University of Kiel, Am Botanischen Garten 11, Kiel, 24118 Germany; 9 Christian-Albrechts-Universität Kiel, Kiel, Germany

**Keywords:** Symbiont, Gonochorism, Embryogenesis, Phagocytosis, Microbiome

## Abstract

**Background:**

Animal development is frequently supported by microbial symbionts that contribute to host nutrition, metabolism, and physiology. While the functional importance of microbiomes in adult hosts is increasingly recognized, the role of symbiotic microbes during gametogenesis and embryogenesis remains poorly understood, particularly in early-diverging metazoans. Sponges represent an ideal system to investigate these processes due to their dense and diverse microbial communities. Here, we examined host-symbiont dynamics across the reproductive cycle of the marine sponge *Halichondria panicea* to assess how microbial communities contribute to reproduction and early development.

**Results:**

Specimens were collected monthly from February to July and classified by reproductive stage using histological analyses. We combined ultrastructural imaging, dual RNA sequencing of host and symbionts, 16S rRNA gene amplicon sequencing, and quantitative PCR to characterize microbial and transcriptional dynamics throughout reproduction. Pronounced shifts in both host gene expression and microbial community composition occurred during early embryogenesis, particularly in May. Transmission electron microscopy revealed nurse cells phagocytosing bacterial aggregates in close proximity to late oocytes, presumably converting them into yolk precursors. This coincided with a significant decline in the abundance of the dominant obligate symbiont, *Candidatus* Halichondribacter symbioticus. Host transcriptomic analyses showed upregulation of immune and phagocytic pathways, including pattern recognition receptors, lectins, and vesicle trafficking components, specifically in females undergoing embryogenesis in May. Concurrently, symbiont gene expression profiles indicated responses to acidic conditions, consistent with exposure to phagosomal environments.

**Conclusions:**

Our results are consistent with intracellular digestion of microbial symbionts during early embryogenesis, potentially to supplement the nutritional requirements of embryogenesis. These findings reveal symbiont phagocytosis as a previously underappreciated nutritional strategy during animal development and highlight the dynamic functional integration of microbiomes into reproductive physiology in basal metazoans.

Video Abstract

**Supplementary Information:**

The online version contains supplementary material available at https://doi.org/10.1186/s40168-026-02473-y.

## Background

Symbiotic associations with microbial communities are fundamental to the biology of many multicellular organisms, providing essential metabolic capabilities and contributing to host physiology, development, and homeostasis. These host-microbe interactions are dynamic and often shift across the life cycle, playing critical roles in both reproduction and ontogeny [1–3]. One of the most striking forms of microbial influence is the modulation of host reproductive compatibility. For instance, in arthropods and nematodes, *Wolbachia* manipulates host reproduction by inducing cytoplasmic incompatibility to affect embryo viability and distort population structure towards female-dominance [4]. In contrast to manipulative strategies, many symbionts serve as essential facilitators of reproductive initiation. Bacterial cues have been shown to trigger the onset of sexual reproduction in several aquatic organisms, including diatoms [5, 6], jellyfish [7], freshwater hydrozoans [8, 9], and sea anemones [10]. In the bobtail squid, symbiotic bacteria are necessary for the proper development of the reproductive organ, demonstrating the profound anatomical and functional influence of microbial partners [11]. Beyond reproductive initiation, symbionts also support gametogenesis and embryonic development through nutritional and physiological contributions. In insects, symbiotic bacteria contribute to reproductive and developmental success by facilitating vitellogenin uptake into oocytes, enhancing larval survival, as seen in bees, and colonizing embryos prior to larval emergence to meet nutritional demands, as observed in beetles [12–14]. Parallel patterns are observed in marine invertebrates; for example, echinoid larvae, shifts in microbial composition prior to the onset of feeding suggest a critical nutritional role of the microbiota during embryogenesis [15].

In sexually reproducing species, mounting evidence indicates that microbial communities often diverge between males and females, influenced by differences in physiology, hormone levels, and reproductive roles [16–19]. In several taxa, including corals [20], mollusks [19, 21], and nematodes [22], sex-specific microbiome profiles have been documented. Although sexual dimorphism in size or habitat may contribute to microbiome divergence, intrinsic reproductive demands (e.g., yolk formation or selection for vertical transmission of microbes) likely play a significant role in female development. Microbial partner selection for vertical transmission may occur at multiple reproductive stages (e.g., oocyte maturation or embryogenesis) when specific patterns of symbiont enrichment are established [18]. For instance, in various insect lineages (e.g., cockroaches, termites, and beetles), specialized bacteriocytes mediate the packaging and delivery of beneficial symbionts to developing oocytes to ensure reliable passage to the next generation [23]. These symbionts can be regulated by lytic pathways and modulated by host developmental and physiological cues [14].

Sponges (phylum Porifera), as early-diverging metazoans, represent a powerful model to investigate conserved mechanisms of host-microbe crosstalk and its modulation across developmental stages. They host remarkably diverse and dense symbiotic microbial communities, which can comprise up to 35% of the sponge biomass [24], and contribute to host metabolism through nutrient cycling, chemical defense, and metabolic exchange [25–27]. In adult sponge tissues, the aquiferous system serves as the primary interface between the sponge and its microbial environment. Bacteria arriving with the filtered water current are captured by choanocytes, which can either digest them intracellularly or transfer them to amebocytes or archaeocytes [28]. Once internalized by archaeocytes, bacteria face divergent fates: digestion for nutritional purposes, long-term retention as symbionts, or release back into the mesohyl extracellular space (reviewed in [29, 30]. The mechanisms by which sponges distinguish between bacteria destined for digestion and those maintained as symbionts remain poorly understood, but likely involve pattern recognition receptors and symbiont-derived molecular signals that modulate the host phagocytic response [30].

Despite the recognized ecological and metabolic importance of sponge-associated microbes, their direct role in host development remains largely unexplored. Functional evidence is currently limited to *Amphimedon queenslandica*, in which vertically inherited symbionts provide essential amino acids required for larval settlement and metamorphosis [31]. Although several studies have described microbial compositional changes across early developmental stages in various sponge species [32–35], investigations into microbiome dynamics linked to reproductive state are only beginning to emerge [36]. Most existing studies provide static snapshots of microbiome composition, often limited to a single life stage [37–39], thus not capturing the full temporal and functional complexity of host-microbiome interactions during reproduction.

Sponges exhibit a relatively plastic sexual strategy; their ancestral stem lineage was likely hermaphroditic, yet many groups have transitioned independently to gonochorism over evolutionary timescales [40]. This shift carries significant ecological implications, as gonochoristic species invest differently in male and female functions. Females, in particular, require additional nutritional resources to produce yolk for their eggs and support larval development. As a result, alternative sources of nutrition, such as symbiont-derived metabolites, may become increasingly important during female reproduction [30]. Recent findings indicate microbiome restructuring during the early stages of oogenesis in female sponges, potentially driven by phagocytosis of symbionts to meet the high energetic demands of yolk formation [36]. Therefore, we hypothesize that symbiont digestion and associated microbial shifts will be most pronounced during the peak of reproductive investment, particularly in females. However, a comprehensive understanding of the contribution of the microbiome to sponge reproduction requires examining microbial dynamics across both pre- and post-reproductive stages. During reproduction, symbionts contained within nurse cells (i.e., cells producing yolk to supply to the gametes), sponge oocytes, and/or embryos face one of two fates: they may be retained intact for vertical transmission to offspring, or they may be phagocytosed and digested to contribute to the yolky nutrient reserves of oocytes and developing larvae [29, 30, 41, 42]. TEM-based studies across multiple sponge species have documented both processes occurring simultaneously, with nurse cells playing a central role in both symbiont provisioning to oocytes and embryos and digestion [42–44]. In addition, oocytes can directly engulf microbes from the mesohyl in the absence of nurse cells [45–47]. Whether phagocytosis of symbionts during embryogenesis primarily serves a nutritional function, regulates symbiont population size, facilitates selective packaging for transmission, or involves a combination of these processes, remains an open question in sponge reproductive biology [29, 30]. Addressing this question requires integrating ultrastructural evidence with molecular data, an approach that has so far been rarely applied in this system.

To address this knowledge gap, we investigated microbial dynamics across the reproductive cycle of the sponge *Halichondria panicea*, a species with a bacterial community that is dominated by the obligate symbiont *Candidatus* Halichondribacter symbioticus (*Rhodobacterales, Alphaproteobacteria*) [48–50]. This bacterium is consistently present across the geographic range of *H. panicea* and is not found to associate with other sponges or to be free-living in the seawater [48]. A highly specific, host-dependent lifestyle is reflected in the genome of *Ca.* H. symbioticus. Specifically, the partially reduced genome of this obligate symbiont has lost some of its ability to regulate transcription and all of its ability for flagellar biosynthesis, is enriched with genes for genomic defense, and has the potential to exchange essential amino acids, vitamins, and sugars with the host [48]. The significance of this symbiont to host fitness is reflected in how it maintains this interaction across generations. *H. panicea* has a maternal pool of *Ca.* H. symbioticus that is provisioned proportionally to reproductive output and allometrically by offspring size, suggesting that it may be closely linked to reproduction [51]. In the present study, we combined ultrastructural analyses, host and microbial transcriptomics (dual RNAseq), quantitative PCR, and 16S rRNA amplicon sequencing to characterize symbiont dynamics in *H. panicea* from February to July, covering all stages of the reproductive cycle. To our knowledge, this is the first study where sponge host and symbiont transcriptomics were performed in parallel and over time. Our results provide evidence for selective symbiont phagocytosis during embryogenesis and uncover host pathways involved in symbiont recognition and digestion, offering novel insights into the functional integration of symbionts into reproductive physiology in a basal metazoan.

## Methods

### Sponge collection and sample preservation

Adults were collected from the breakwaters at Schilksee Strandbad (Kiel, Germany; 54.424772, 10.175033) during the known reproductive period in the area for *H. panicea* (Barthel 1986; Witte, et al. 1994). Individuals (average 15 individuals/month, Table 1) were collected monthly from February to July of 2022 by gently removing sponges from boulders using a paint scraper. All individuals were kept fully submerged in seawater and then transferred to the GEOMAR Helmholtz Centre for Ocean Research (Kiel, Germany). Tissues from individuals were preserved in (i) 2.5% glutaraldehyde to identify host reproductive stages by histology, (ii) 99% ethanol to qualitatively and quantitatively describe the bacterial community by amplicon sequencing and qPCR, and (iii) RNAlater to characterize the host-symbiont crosstalk during the reproductive cycle by metatranscriptomics. Samples for histology were stored at 4 ºC, for amplicon sequencing and qPCR were kept at − 20 ºC, and for metatranscriptomics were stored at − 80 ºC.

**Table 1:** Summary table of the *Halichondria panicea* samples used in the study for each month

Month	Reproduction	Stage	*N*^*(*A)^	*N*^(B)^	*N*^(C)^
February	Reproductive	Early oocytes	6	6	0
	Non-reproductive	Non-reproductive	9	9	0
March	Reproductive	Mid oocytes	5	5	4
		sperm	2	2	0
	Non-reproductive	Non-reproductive	8	6	2
April	Reproductive	Late oocytes	8	8	4
		sperm	4	4	4
	Non-reproductive	Non-reproductive	3	1	
May	Reproductive	Late oocytes	5	5	1
		Late oocytes/ early and mid embryos	3	3	3
		Early embryos	3	3	1
		sperm	4	4	3
	Non-reproductive	Non-reproductive	1	1	0
June	Reproductive	Late embryos/larvae	6	6	3
	Non-reproductive	Non-reproductive	9	7	3
July	Non-reproductive	Non-reproductive	14	14	0
Total			90	84	28

The reproductive stage of the samples was verified according to the TEM observations.

^A^Number of samples used for the assessment of the reproductive biology of *H. panicea*

^B^Number of samples used for the amplicon study

^C^Number of samples used for the host and symbiont community transcriptome

### Histology

Siliceous spicules were removed by bathing sponge tissue fragments (0.5 cm^2^) in 5% hydrofluoric acid overnight and were rinsed with distilled water 2–3 times. Tissues were then processed for either light microscopy or transmission electron microscopy (TEM). For light microscopy, tissues were dehydrated through an increasing ethanol series (30 min × 3 each) and were embedded in paraffin overnight after a 5 min rinse in xylene. Paraffin blocks were sectioned at 5 μm with an HM 325 rotary microtome (ThermoFisher-Scientific) and stained with a standard hematoxylin and eosin protocol. Serial sections were mounted in slides with DPX and observed with an Olympus microscope (BX43) with a UC50 camera at the Museo Nacional de Ciencias Naturales de Madrid (CSIC). The size (i.e., maximum diameter) of 20–30 reproductive elements (oocytes, spermatic cysts, embryos, and larvae) was quantified for each sample.

For TEM, sponge tissues were rinsed in PBS and 0.6 M NaCl, post-fixed in 2% osmium tetroxide in 0.4 M PBS and potassium-ferrocyanide for 1–2 h at 4 ºC, and thoroughly rinsed in distilled water afterwards. An increasing ethanol series was used post-staining to dehydrate the tissue for further embedding in epoxy resin. Ultrathin sections (65 nm) were obtained from epoxy blocks with an Ultracut ultramicrotome (Reichert-Jung) and used UranyLess (Electron Microscopy Sciences) and lead citrate to stain. Ultrathin sections were observed on gold-coated grids with a JEOL 1400 Plus transmission electron microscope at the imaging facilities at the Centro Nacional de Microscopía Electrónica (CNME) in Spain.

### Amplicon sequencing and bioinformatic analyses

Total DNA was extracted from all samples, as well as DNA blanks (*n* = 3), according to the manufacturer’s protocol for the DNeasy® Blood & Tissue Mini Kit (Qiagen). Total DNA was quantified using the dsDNA HS Assay Kit for the Qubit Fluorometer (Thermo Fisher Scientific) following the manufacturer’s protocol. Total DNA was diluted to 0.5 ng/µL for amplicon sequencing. Samples for amplicon sequencing (V3/V4 region of the 16S rRNA gene) were sent to the Microbiome Laboratory of the Institute of Clinical Molecular Biology, where libraries were prepared and Illumina MiSeq sequencing (v3, 2 × 300 bp paired-end reads) was performed at the Competence Center for Genomic Analysis (CCGA; Christian-Albrechts University of Kiel, Germany).

Raw 16S rRNA gene sequences were processed using the UPARSE pipeline [52]. Briefly, primer sequences were removed and overlapping paired-end reads were merged using *-fastq_mergepairs,* allowing for a maximum of 10 bp differences. After quality check and de-replication, unique read sequences were detected using the *fastx_uniques* command. Denoising (error-correction) of amplicons was performed using *unoise3* [52], which removed chimeras, reads with sequencing errors, PhiX, and low-complexity sequences due to Illumina artifacts, and generated ZOTUs (“zero-radius” operational taxonomic units) with 100% identity sequences. Taxonomic assignment was done with SINA v1.2.11 using SILVA 138 database [53]. Sequences with low alignment quality (< 75%) or unclassified as well as sequences identified as mitochondria or chloroplasts were removed from the analysis. The initial ZOTU table was rarefied, with the minimum threshold being set at the sample containing the fewest reads (4863 reads) and rarefaction curves are provided as Supplementary Fig. S1. This rarefaction process was performed using the *rrarefy* function from the R package *vegan* v2.5–6 [54]. The rarefied ZOTU table was subsequently employed for conducting alpha diversity analyses. Moreover, the raw counts table was converted to a relative abundance ZOTU table and utilized for characterizing the microbial community and conducting beta diversity analyses.

A distance-based multivariate analysis was conducted of the microbial communities within the sponge samples at the ZOTU level using the *vegan* v2.5–6 [54] in R [55]. A Bray–Curtis dissimilarity distance matrix based on the log2-transformed relative abundance ZOTU table was used to visualize the patterns of bacterial community structure across time in a principal coordinates analysis (PCoA) using the *cmdscale* function of the *vegan* R package. We first tested the effect of sampling time (months) on the structure of microbial communities with non-parametric permutational analysis of variance (PERMANOVA) using the *adonis* function using 999 permutations and a cut-off of 0.05 *p*-value. Following the evaluation of a month’s impact on microbial communities, we created distinct subsets for each month to examine the influence of the reproductive state on each subset. For each month, we calculated Bray–Curtis dissimilarity distance matrix and visualized the ordinations through PCoAs, as described above. The effect of reproduction (reproductive vs. non-reproductive) and reproductive state (oocytes, sperm, embryo, larvae, or non-reproductive) on the sponge microbiomes was tested using the *adonis* function. Moreover, generalized linear models were performed in the R package *EdgeR* v.3.26.8 [56] to discern variations in the abundance of particular microbiome ZOTUs across different reproductive stages using *p*-value of 0.05 as a threshold. A rarefied dataset was used to compute alpha diversity indices, specifically Richness and Shannon, within the *vegan* package. To elucidate the disparities in alpha diversity across months and reproductive stages, we conducted an analysis of variance (ANOVA) followed by pairwise comparisons using TukeyHSD test to pinpoint significant differences in alpha diversity.

### Quantification of total bacterial and *Ca*. H. symbioticus symbiont abundance

The total bacterial community abundance was quantified using eubacterial primers for the 16S rRNA gene (F: TGCATGGYTGTCGTCAGCTCG; R: CGTCRTCCCCRCCTTCC; [57]), which amplifies a 141 bp fragment. We tested the in silico affinity of these primers, and found that they had very low affinity to Rhodobacterales (8%) but high to most other abundant taxa of the host microbiome, recovering 76% of the taxa in the SILVA rRNA database. The obligate symbiont of *H. panicea*, *Ca*. H. symbioticus, was quantified using species-specific primers for the 16S rRNA gene (F: CGCGGATGGTAGAGATACCG; R: TGTCCCCAACTGAATGCTGG; [49]), which amplifies a 148 bp fragment.

A standard curve was prepared to calculate the number of 16S rRNA gene copies in each sample by running a 50-µL PCR reaction. This product was then verified using gel electrophoresis and then isolated from the gel following the manufacturer's protocol for the NucleoSpin Gel and PCR Clean-up kit (Marcherey Nagel). Total DNA was then quantified following the manufacturer’s protocol for the dsDNA BR Assay Kits for the Qubit Fluorometer kit (Thermo Fisher Scientific). We then used the quantified DNA, elution volume, and fragment length to calculate the number of copies to calculate the number of 16S rRNA gene copies, upon which we then prepared a tenfold dilution series from 10^–9^ to 10^–3^ ng/µL of DNA to be used as a standard curve.

Samples from individual adult sponges were randomly assigned across several 96-well plates, with all three replicates for eubacteria and *Ca.* H. symbioticus being on the same plate. Each qPCR reaction (totaling 20 µL) was performed in triplicate using 4 µL of template, 10 µL of the Maxima SYBR Green qPCR Master Mix (Thermofisher), 0.1 µL of forward and reverse primers (total of 250 nM), and 5.8 µL of DNAase and RNAase-free water. The following cycle parameters were used on a CFX Connect Real-Time PCR Detection System (Bio-Rad) thermo cycler: 96 °C for 2 min, followed by 40 cycles of 94 °C for 30 s, 60 °C for 30 s, and 72 °C for 60 s, and a melting curve analysis was conducted by increasing temperature from 60 °C to 95 °C every 5 s. Absolute copy number was then calculated according to the standard curve. Quantitative estimates for each adult sponge were normalized by log transformation. Log-transformed data for non-reproductive, reproductive females, and reproductive males were compared within each month using analysis of variance (ANOVA) with a priori pairwise comparisons using the *emeans* package in R.

### Metagenome-assembled genome of the local *Ca*. H. symbioticus strain

We collected a separate individual *H. panicea* sponge in September 2020 from the Schilksee Strandbad using the same methods described above. Tissue was dissected from one individual (i.e., biological replicate), purged of seawater, and frozen by liquid nitrogen. DNA extraction and computational methods were performed by Wellcome Sanger Institute following Aquatic Symbioses Genomics standard procedures and pipelines, for example as published in Pita et (2025) [58].

Raw reads were trimmed and quality checked using BBTools (BBDuk v.38.96) and FastQC, respectively. The metagenome was assembled using Flye [59] and binned using MetaBAT2[60], MaxBin [61], bin3C [62], and MetaTOR [63]. The resulting set of bins from these binning algorithms was then optimized and refined using MAGScoT [64]. PROKKA [65] was used to identify tRNAs and rRNAs in each bin, CheckM [66] was used to assess bin completeness/contamination, and GTDB-TK [67] (v. 214) was used to taxonomically classify bins. Taxonomic replicate bins were identified using dRep [68]. The final bin set was filtered for bacteria and archaea. All bins were assessed for quality following MIMAG standards [69]. Genome bins with ≥ 50% completeness and ≤ 10% contamination were classified as metagenome-assembled genomes (MAGs), further designated as high-quality MAGs (≥ 90% completeness and ≤ 5% contamination) or medium-quality MAGs (≥ 50% completeness and ≤ 10% contamination). Bins that did not meet the ≥ 50% completeness threshold were retained as “binned metagenomes”. Bakta [70] was then used to identify coding regions, annotate, and construct metabolic pathways for the local *Ca*. H. symbioticus MAG, which meets the criteria for a high-quality MAG [71].

### Metatranscriptomics

Total RNA was extracted from 28 selected samples (Table 1) using the RNeasy Plus Mini kit (Qiagen) and a protocol tailored for *H. panicea* [72]. Total RNA was quantified using the RNA BR Assay Kits for the Qubit Fluorometer (Thermo Fisher Scientific). Ribosomal RNA was then depleted using the Ribo-Zero Plus kit (Illumina) and library preparation was performed without a poly-A enrichment (to keep both host and microbiome reads) using the TruSeq stranded mRNA kit (Illumina). Illumina NovaSeq 6000 (S1; 2 × 150 bp paired-end reads) was performed at the CCGA at the University of Kiel (Kiel, Germany).

Illumina adapters were removed using Trimmomatic [73] and rRNA reads were removed using SortMeRNA v2.1 [74] with the SILVA 119 Ref NR 99 database [53]. Reads were de novo assembled with the Trinity software v2.0.6 [75], using default settings for strand-specific, paired-end reads, and a minimum transcript length of 150 bp. Transcript abundance for each sample was estimated with RSEM v1.3.1 [76], which implements Bowtie [77] to map reads back to the reference metatranscriptome and calculates the expected counts (*i.e.*, number of reads mapped) and the normalized trimmed mean of *M* values (TMM). We looked for differential expression (DE) of genes between reproductive and non-reproductive or between male and female specimens within each month, to account for variability of sponge and microbial load with *EdgeR* using a *p*-value threshold of 0.05 [56], separately. We used several strategies to annotate the metatranscriptome and separate host and microbial transcripts. The reference metatranscriptome was blasted against Swiss-Prot [78] and against the genome of the sponge host (National Center for Biotechnology Information; ref: GCA_963675165.1) and Metagenome-Assembled Genome (MAG) of the local *Ca*. H. symbioticus strain (See section Metagenome‑assembled genome of the local *Ca.* H. symbioticus strain), the main symbiont of the sponge. In addition, the metatranscriptome was also uploaded to the IMG/M [79] for gene annotation [80–82] and FANTASIA for Gene Ontology (GO) enrichment [83].

Transcripts associated with the host were annotated using BLAST2GOPRO [84] to obtain the GO annotations. DE genes were implemented in shinyGO [85] to perform GO enrichments. Only the most relevant categories for each condition containing more than 5 genes were plotted in each pathway, considering the value for Enrichment FDR (multiplied by 10 for better visualization) and avoiding duplication of genes in categories. For the microbial transcripts, GO terms obtained from FANTASIA from DE genes were visualized with REVIGO [86], but only those previously reported in bacteria were plotted. Finally, clusters of co-expressed genes were obtained with *Mfuzz* [87] using soft clustering. These clusters were assessed separately for both sponge transcripts and *Ca.* H. symbioticus transcripts. Then, genes in each cluster of interest were inspected in shinyGO for enrichments in KEGG pathways using the same shinyGO procedure and statistical settings as described above for the GO enrichment (Enrichment FDR threshold, minimum of 5 genes per category).

## Results

### Host dynamics during reproduction

#### Reproductive biology of *Halichondria panicea*

**Fig. 1:**
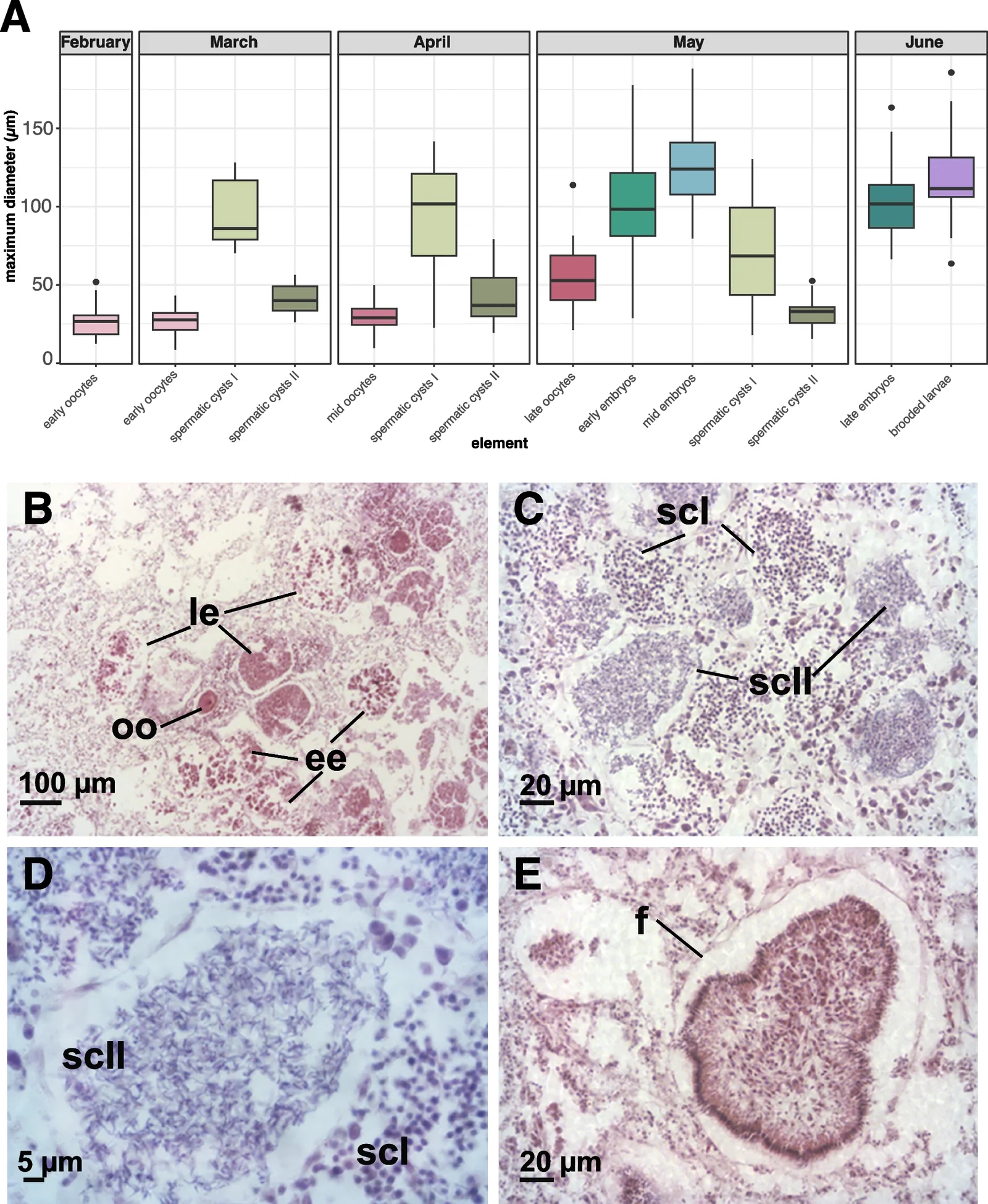
Gametogenesis of *H. panicea* and structure of its reproductive elements: **A** Box plot of the size (maximum diameter) of the reproductive elements (oocytes, embryos and spermatic cysts) from February to June. **B** Oocytes (oo), early embryos (ee) and late embryos (le) in a female individual from April. **C** Primary spermatic cysts (scI) with spermatocytes and secondary spermatic cysts (scII) with spermatids in a male individual from March. D) Fully developed secondary spermatic cyst (scII) with sperm in a male individual from April. E) Pre-competent larvae surrounded by a follicle (f) in a female from June

This *Halichondria panicea* population was consistently gonochoristic, viviparous, and had a 1.75 female-to-male ratio. Approximately 40% of this population initiated reproduction in February and March. Peak reproductive activity (~ 65% of the population) coincided with the apex of spermatogenesis in April, after which the percentage of individuals incubating reproductive elements returned to ~ 40% in June (Supplementary Fig. S2). The initial oocytes in February had an average maximum diameter of 25 µm, while oocytes in May had an average maximum diameter of 50 µm (Fig. 1A, B; Table 1). Spermatogenesis initiated in March and continued through May. The process was quick, whereby primary (i.e., spermatic cysts I; spermatogonia and primary/secondary spermatocytes) and mature (i.e., spermatic cysts II, spermatids and mature sperm) spermatic cysts were observed across all months (Fig. 1A, C–D; Table 1). Spermatic cysts II were consistently smaller and more compact than spermatic cysts I but contained more spermatocytes (Fig. 1A, C–D; Table 1). Finally, embryogenesis was limited to May and June, with early (100 µm in average diameter) and mid-embryos (125 µm in average diameter) developing in May, while late embryos and pre-competent larvae (105 µm and 110 µm of maximum diameter in average, respectively) were observed in June (Fig. 1A–E). Larvae were small and compact parenchymellae, and were completely surrounded by a follicle layer (Fig. 1E).

### Differential transcript expression of the host sponge

**Fig. 2:**
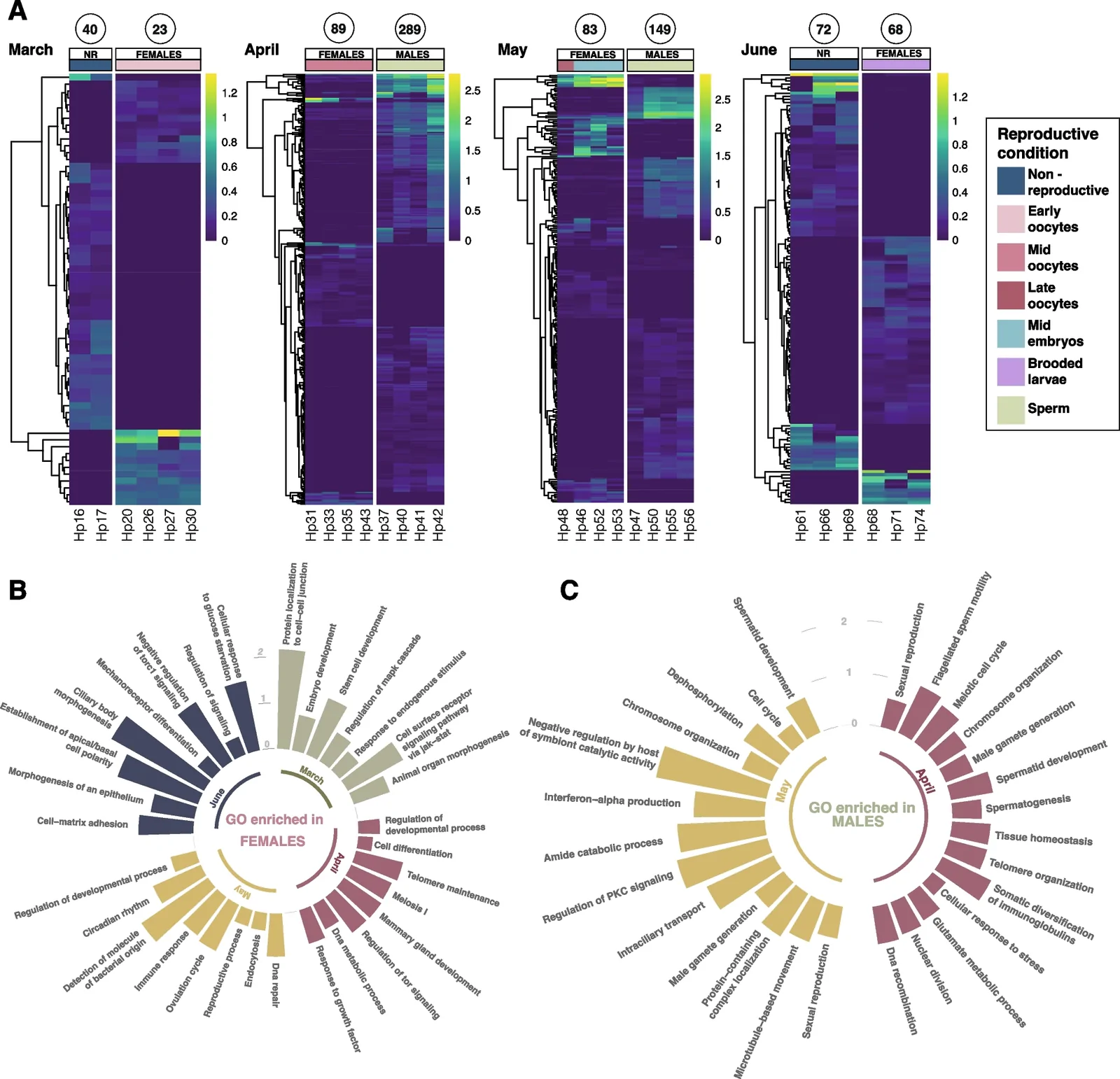
Host transcriptome: **A** heatmaps of the differentially expressed (DE) transcripts between reproductive conditions for each month. The number of DE transcripts is indicated for each heatmap group. The temperature bar corresponds to the log-transformed expression of each transcript. **B** Gene Ontologies (GO) enriched for females (values of enrichment FDR) in each month. **C** Most abundant Gene Ontologies (GO) enriched for males (values of enrichment FDR) in each month

We obtained a total of 580,582,954 raw reads, but after the quality filtering and the removal of ribosomal reads, we retained only 15% of them for the assembly (Supplementary Table S4A–B). The total metatranscriptome comprised 328,159 transcripts with an N50 of almost 1500 bp, of which 75,373 were identified as belonging to the host sponge (Supplementary Table S4A, C). The BUSCO values for the host transcriptome were very high, recovering 93.5% of the metazoan cassette (Supplementary Table S4A). From the retained reads, approximately 65% from each library were mapped to the reference transcriptome and used for the analysis of gene expression (Supplementary Table S4B, C). Due to seasonal variation, DE transcripts were compared only between reproductive states within each month (2A). In March, the transcriptional differences between females with early oocytes and non-reproductive individuals were small (23 vs. 40 DE transcripts in each), but such differences were greater in the comparisons between male and female individuals for April and May (89 in females vs. 289 in males in April, and 83 in females vs. 149 in males in May; Fig. 2A, Supplementary Table S1). In June, the transcriptional differences revealed 72 differentially upregulated transcripts in females incubating larvae and 68 differentially upregulated transcripts in non-reproductive individuals (Fig. 2A, Supplementary Table S1). As for female individuals, the enriched Gene Ontologies (GO) of the differentially upregulated transcripts varied across months, reflecting the development of gametogenesis and the impact on the entire physiology of the sponge (Fig. 2B). In March, the DE transcripts were enriched in GO categories like cellular embryo development, stem cell development, and regulation of MAPK cascade, among others (Fig. 2B). For instance, *Scribble* (*SCRIB*) is responsible for different aspects of polarized cell differentiation during embryo development and this was upregulated in females (Supplementary Table S2). Other DE genes in March in the females were *syntaxin 5* (*STX5*) and *E3 ubiquitin-protein ligase NRDP1* (*RNF41*), involved in vesicle trafficking (Supplementary Table S2).

In April, enriched GO terms in females were more indicative of the genetic process going on, including the first phase of meiosis, and other GO categories like cell differentiation, response to growth factor, telomere maintenance, and regulation of tor signaling (Fig. 2B). Other genes upregulated in April in female samples were *hypoxia-inducible factor 1-alpha* (*HIF1A*) and *xanthine dehydrogenase/oxidase* (*XDH*) (Supplementary Tables S1 and S2). These genes are functionally linked through hypoxia and oxidative stress pathways, given that *HIF1A* is activated under hypoxia and can modulate genes affecting redox state and *XDH* activity produces ROS, which can influence hypoxia signaling. Other genes upregulated in April females were related to telomere maintenance, including *telomerase protein component 1* (*TEP1*) and *nuclear valosin-containing protein-like* (*NVL*). Both are components of, or interact with, the telomerase ribonucleoprotein complex (*TERC/TERT/TEP1*), whose activity in oocytes and ovarian stem cells is essential for preserving genomic stability and replicative capacity.

In May, there were GOs related to gametogenesis in females, such as ovulation cycle, reproductive process, circadian rhythm, and DNA repair, but also there was a greater enrichment in GOs related to immune response and detection of a molecule of bacterial origin (Fig. 2B, Supplementary Table S2). The genes involved in the latter categories were mainly *TNF receptor-associated factor 4* (*TRAF4*) and *lipopolysaccharide-binding protein* (*LBP*) (Fig. 2B, Supplementary Table S2). In May, females overexpressed one paralog of V-type proton ATPase 16 kDa proteolipid subunit (*VATL*), out of the 11 paralogs, being the other 10 non-DE. Moreover, lectin (*LECA*) was consistently differentially expressed in females and showed its peak of expression during the cycle (Supplementary Table S1).

Finally, in June, when embryonic development progressed, females showed enrichment of GOs linked to larval morphogenesis, including establishment of apical/basal cell polarity, epithelium and ciliary body morphogenesis, and mechanoreceptor differentiation, as well as signaling processes such as the negative regulation of torc1 signaling (Fig. 2B, Supplementary Table S2). Within the latter category, hamartin (*TSC1*) and *KICSTOR complex protein SZT2* (*STZ2*) were significantly upregulated in the females brooding larvae (Supplementary Table S2). Consistent with the fact that pre-competent larvae are fully ciliated, we also found enrichment in GOs related to cilium morphogenesis including *jagged 1* (*JAG1*) (Supplementary Table S2).

For males, the GO enrichments DE transcripts compared to females varied between April and May (Fig. 2C). In April, enriched GO categories were primarily associated with sperm formation, including sexual reproduction, spermatogenesis, male meiosis I, male gamete generation, and spermatid development (Supplementary Table S2). Key genes in these categories included *MutS protein homolog 5* and* 6* (*MSH5* and *MSH6*), *Forkhead box protein J3* (*FOXJ3*), *Meiotic recombination protein DMC1/LIM15 homolog* (*DMC1*), among others (Supplementary Table S2). Additional enrichments in April were related to tissue homeostasis and glutamate production, the latter being important as glutamate serves as a precursor for glutathione, a potent antioxidant that protects sperm from oxidative stress caused by germ-cell-derived reactive oxygen species (ROS) (Fig. 2C, Supplementary Table S2). Genes annotated in this GO with prominent roles were ornithine aminotransferase (*OAT*) and *formimidoyltransferase-cyclodeaminase* (*FTCD*) (Supplementary Table S2). In May, males continued to show enrichment in similar reproductive GOs, but also in categories related to interferon alpha production, dephosphorylation, and negative regulation by host of symbiont catalytic activity (Fig. 2C, Supplementary Table S2), with upregulated genes including *Ras-related protein Rab-9A* (*RAB9A*), and *protein phosphatase ppm-1.A* (*PPM1A*) (Supplementary Table S2).

### Microbiome dynamics during reproduction

#### *H. panicea *associated microbial diversity

**Fig. 3:**
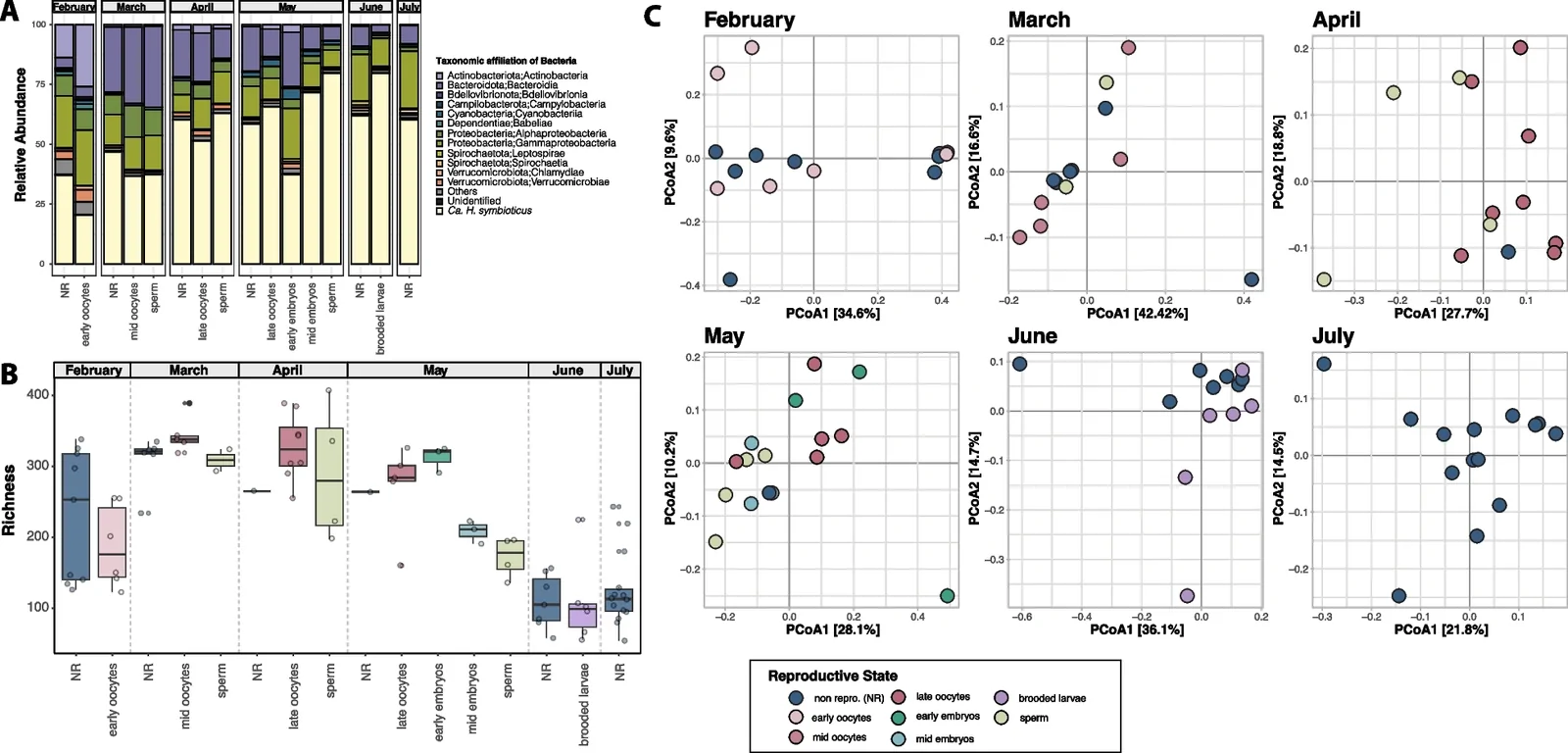
*H. panicea* microbiome. **A** Barplots showing the top 15 taxa at class level for each month and reproductive condition. Abundance of the dominant Ca.H. symbioticus is shown as a separate entity. All the other taxa are grouped as “others”. **B** Boxplot showing the richness for each month separated by reproductive state. **C** Separated PCoAs for each month colored according to reproductive state. Variation explained by the first two axes is indicated as %

We identified a total of 3270 ZOTUs representing 40 different prokaryotic phyla and 91 different classes (Supplementary Table S3A, B). *Proteobacteria* was the most abundant phylum, with an average relative abundance of 70.9%, followed by *Bacteroidota* (12.8%) and *Cyanobacteria* (6.5%). *Ca.* H. symbioticus (ZOTU1), the obligate symbiont of *H. panicea*, was the most dominant taxon with an avgRA of 54.2% (Fig. 3A, Supplementary Table S3C). Its relative abundance varied across the different months (*Kruskall-Wallis* test, *p*-value < 0.001), ranging from an average relative abundance of 30% (± 28% SD) during February to an average relative abundance of 70% (± 23% SD) in June. Generally, *Ca*. H. symbioticus increased in relative abundance from February to June, contrasting with the pattern observed for community richness (Fig. 3A, B). However, within each month, the relative abundance of this symbiont did not differ significantly between reproductive stages.

Alpha diversity values for *H. panicea* exhibited notable variations across different months, with significant differences identified in Richness (Fig. 3B, 32E) and Shannon diversity (Supplementary Fig. S3C, D) indices (ANOVA, *p* < 0.001, Supplementary Table S3D, Supplementary Statistics). Separate analyses for each month were conducted to evaluate the influence of the reproductive state on microbiome alpha diversity. Only sponges collected in May showed statistically significant differences for Richness and Shannon between the different reproductive states (Table 1, Supplementary Statistics, Fig. 3B, Supplementary Fig. S3C).

Regarding beta diversity, significant compositional differences between months were detected (*adonis*, *p* < 0.001, Supplementary Statistics, Supplementary Fig. S3B). While the first PCoA axis separated the samples according to their sampling date, which explained 27% of the variation, the second PCoA axis separated some February samples. This separation is coincident with the presence of *Actinobacteria* in some of those samples (Supplementary Fig. S3A). Separate PCoAs were performed for each month to further assess the effect of reproductive stage on the ordination of bacterial communities (Fig. 3C). Nonetheless, differences according to the reproductive state were not found in any of the months (Fig. 3C, Supplementary Statistics).

The differential abundance (DA) analysis, which compared reproductive and non-reproductive individuals across the entire dataset, revealed that 33 ZOTUs were significantly enriched in reproductive individuals, including those with oocytes, sperm, embryos, and larvae, when compared to their non-reproductive counterparts (Supplementary Table S3E). Conversely, only 10 ZOTUs were found to be more enriched in non-reproductive individuals (Supplementary Table S3E). However, it is essential to note that the overall average relative abundance of these ZOTUs remained below 2%, indicating a minor contribution to the microbiome composition. Results of this overall comparison should be interpreted with caution due to the effect of sampling time on the sponge composition; thus, separate analyses for each month were performed. Only specific comparisons in March, April, and May lead to significant results in terms of the number of DA ZOTUs (Supplementary Table S3E). Nonetheless, none of these DA ZOTUs accounted for more than 1% in the average relative abundance of the microbial community, suggesting little effect of the reproductive stage in the overall composition in terms of relative abundance.

### Quantitative assessment of the bacterial community

**Fig. 4:**
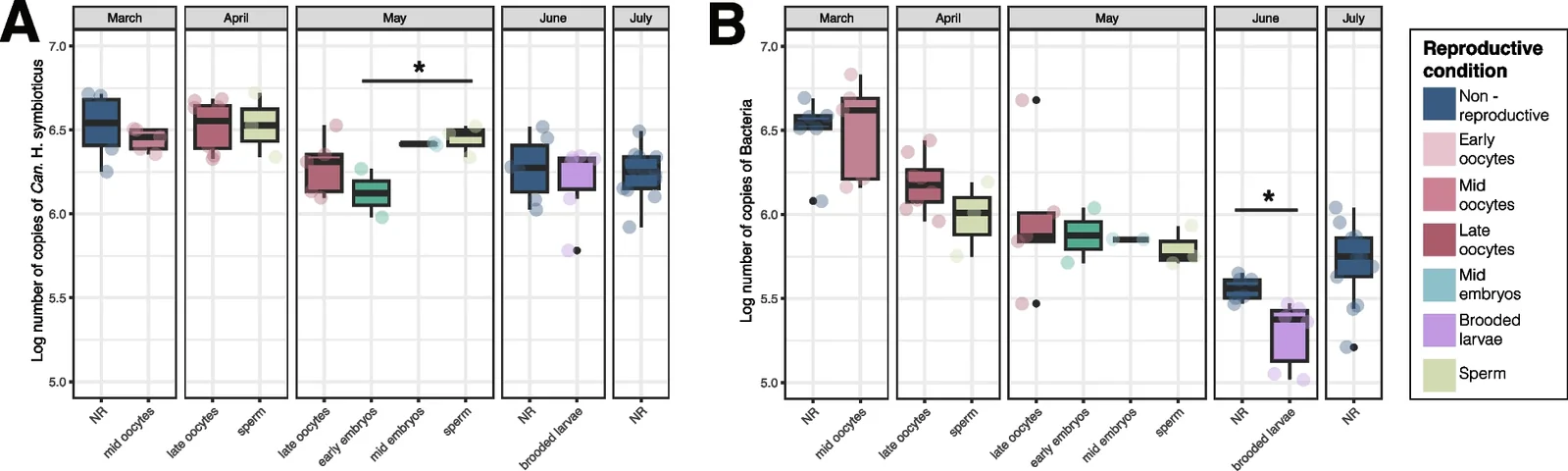
Quantitative assessment of *H. panicea* bacterial community (**A**–**B**) qPCR results of the total abundance of *Ca*. H. symbioticus (**A**) and total bacteria (**B**) along the months for each reproductive condition. (*) Indicates significant differences between reproductive conditions

Quantitative changes in the bacterial load along the reproductive cycle of *H. panicea* for *Ca.* H. symbioticus (Fig. 4A) and total bacteria (Fig. 4B) were assessed among reproductive conditions within each month. Overall, the abundance of *Ca.* H. symbioticus remained stable across the reproductive period and showed the greatest variation among reproductive conditions in May (Fig. 4A, Supplementary Table S7). In contrast, total bacterial abundance decreased from March to June, followed by a slight increase in July (Fig. 4B). Given the poor affinity of primers for the Rhodobacterales 16S rRNA gene*,* the quantification of total bacteria (Fig. 4B) excludes that of the main symbiont, and therefore quantifies the rest of the microbial community. We tested for significant differences between reproductive conditions within each month and found that while the quantity of total bacteria only varied significantly between the non-reproductive individuals and those with brooding larvae in June (one-way ANOVA, *p* = 0.021, Fig. 4B; Supplementary Table S7), that of *Ca.* H. symbioticus varied significantly between females with early embryos and males in May (one-way ANOVA, *p* = 0.03, Fig. 4A).

### Differential expression of the microbial community

**Fig. 5:**
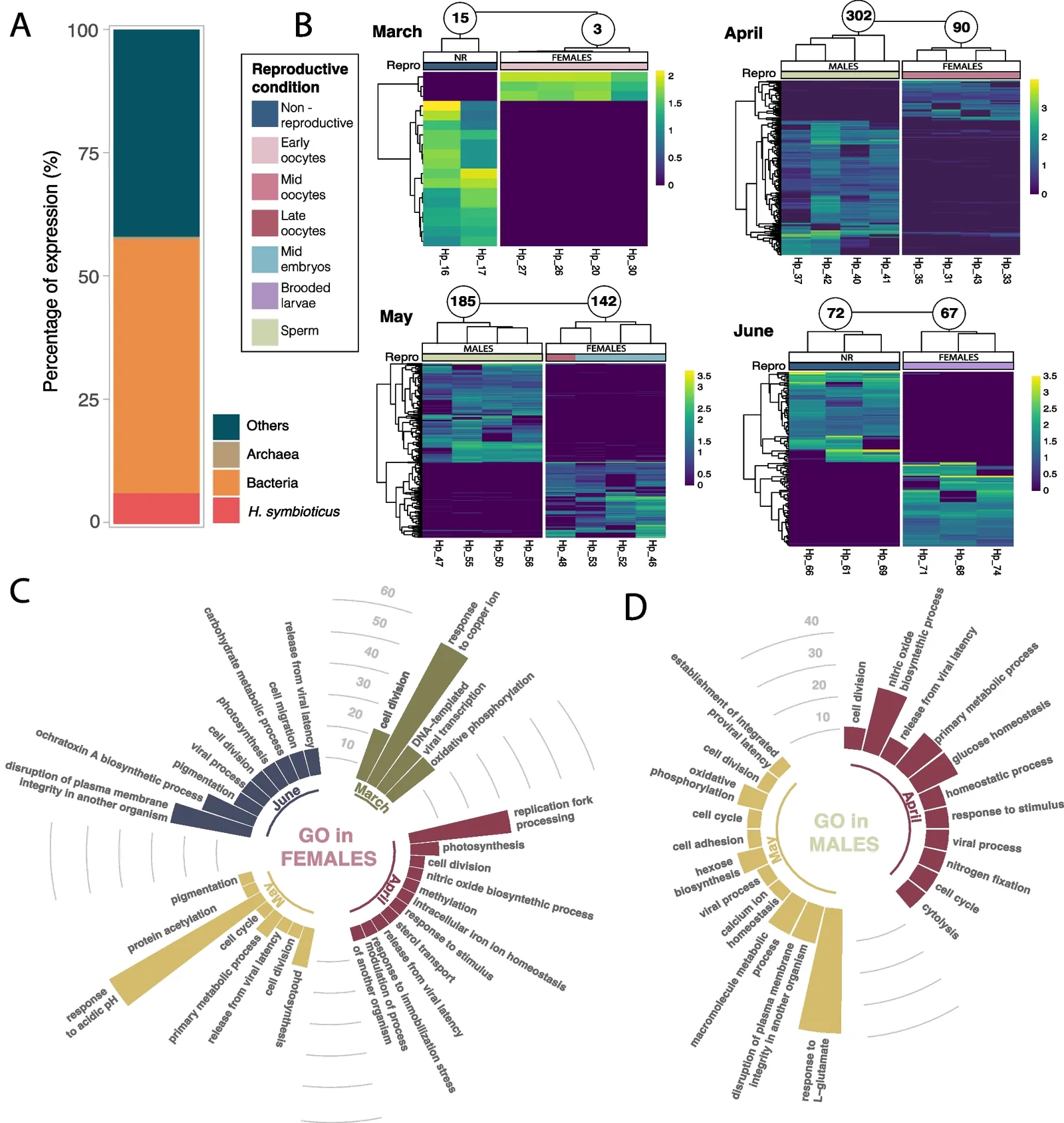
*H. panicea* microbiome expression. **A** Percentages of the total expression from microbes belonging to archaea, bacteria or the main symbiont. “Others” include fungi, algae and virus. **B** Heatmaps of the Differentially expressed (DE) microbial transcripts between reproductive conditions for each month. The number of DE transcripts is indicated in each heatmap cluster. The temperature bar corresponds to the log-transformed expression of each transcript. **C** Most abundant Gene Ontologies (GO) enriched for females in each month with their average expression. **D** Most abundant Gene Ontologies (GO) enriched for males in each month with their average expression

From the 328,159 transcripts in the reference metatranscriptome, 58,940 were annotated as microbial (Supplementary Table S4A, D, i.e., bacteria, archaea, fungi, algae, or viruses). Among these, 9390 were identified as bacteria and 3,090 belonged to the main symbiont *Ca*. H. symbioticus (Fig. 5A, Supplementary Table S4A). On average, transcripts from *Ca.* H. symbioticus accounted for 5% of those identified as microbial and accumulated 7% of the total microbial transcript expression in counts (Fig. 5A; Supplementary Table S4A).

Similar to host gene expression patterns, transcriptional differences of the entire microbial community between reproductive states were more pronounced in the comparisons between males and female individuals in April and May (Fig. 5B, Supplementary Table S5). In March, differences between females and non-reproductive individuals were minimal, with only 15 and 3 DE transcripts, respectively (Supplementary Table S5). However, in April and May, the female vs. male comparisons yielded the highest number of DE transcripts, with 392 and 327, respectively (Supplementary Table S5). By June, transcriptional differences in the microbial community were again modest, with 67 upregulated transcripts in females incubating larvae and 72 upregulated transcripts in non-reproductive individuals (Fig. 5B, Supplementary Table S5). However, none of the DE transcripts between females and males were mapped to the *Ca.* H. symbioticus genome (Supplementary Table S5).

Throughout the female reproductive cycle, enriched Gene Ontologies (GO) of the differentially upregulated transcripts in female individuals were different for each month (Fig. 5C, Supplementary Table S6). In March, the most highly expressed GO category was related to the response to copper ions. By April, DE transcripts were enriched in GO terms associated with replication fork processing and photosynthesis. In May, the most upregulated GO category was response to acidic pH, while in June, the highest-expressed GO terms were related to plasma membrane integrity disruption and ochratoxin A biosynthesis (Supplementary Table S6).

For males, GO enrichment patterns of the upregulated transcripts in male individuals in April and May were related to different processes (Fig. 5D). In April, DE transcripts were predominantly linked to nitric oxide biosynthesis, primary metabolic processes, and glucose homeostasis. By May, DE genes were primarily associated with the response to L-glutamate (Fig. 5D, Supplementary Table S6).

### Interplay between host and its symbiont community

#### Ultrastructural evidence of symbiont phagocytosis

**Fig. 6:**
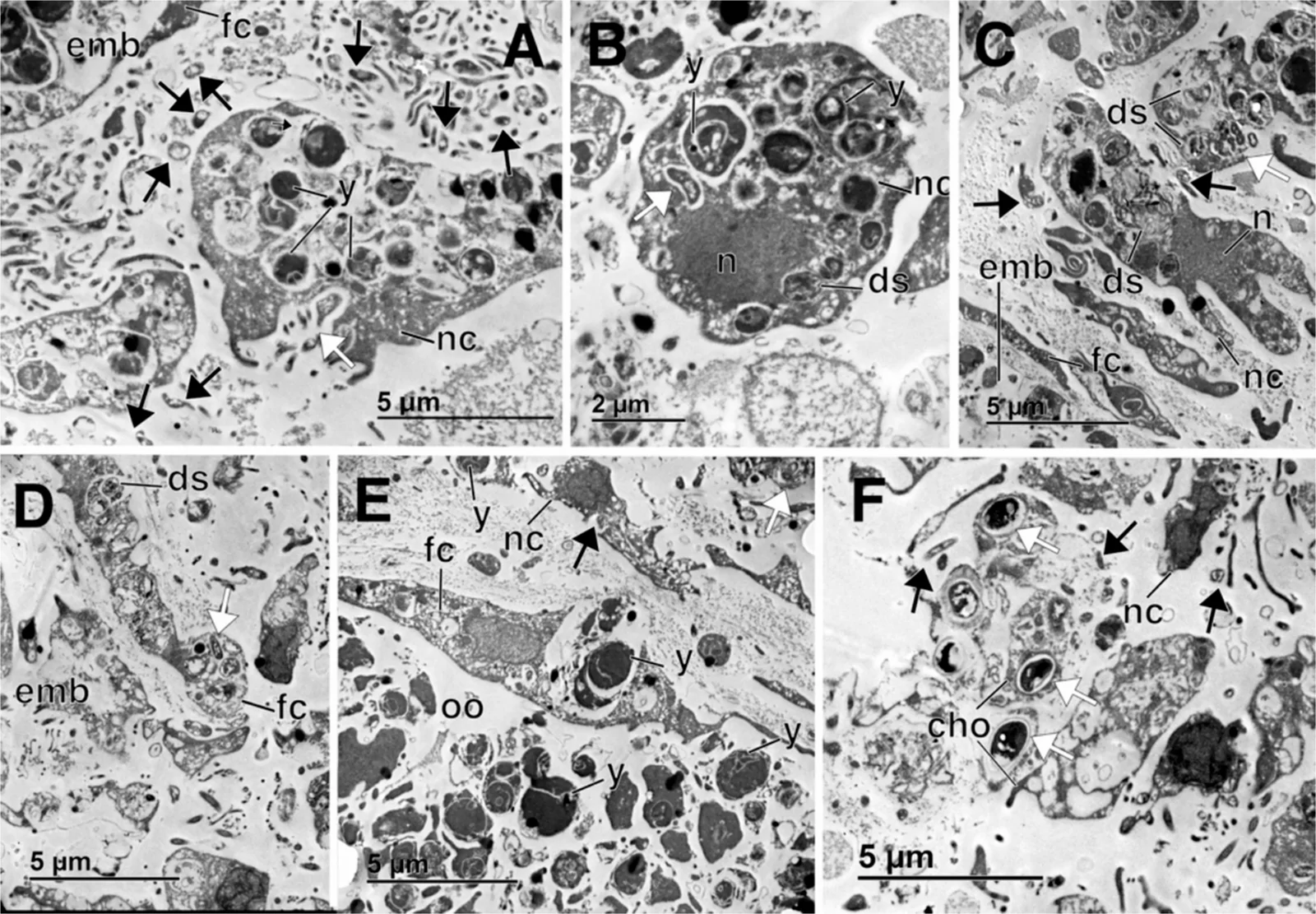
TEM images of female individuals from May. **A** Mesohyl of a female sponge with developing embryos (emb) enveloped by follicle cells (fc) and surrounded by nurse cells (nc) phagocytizing putative symbiotic rod-shaped bacteria (indicated with white arrows). Note the putative symbiotic rod-shaped bacterial cells aggregated in the vicinity of the embryo (black arrows). **B**–**C** Nurse cells (nc) with a non-nucleolated nucleus (*n*) showing putative symbiotic bacteria phagocytosed in a vesicle (white arrow), vesicles with evidence of bacterial digestion (ds), and yolk resulting from bacterial digestion (*y*). Note the vicinity to an embryo (emb) enveloped by follicle cells (fc) and the putative symbiotic bacterial cells (black arrows) lying in the collagenous mesohyl. **D** Follicle cell (fc) enveloping an embryo (emb) showing putative symbiotic bacteria (white arrow) phagocytosed in a vesicle, and vesicles with digested bacterial cells (ds). **E** Mesohyl of a female sponge containing late oocytes (oo) and surrounded by follicle cells (fc) containing yolk platelets (*y*). Note the bacterial cells being digested by nurse cells (white arrows). **F** Choanocyte chamber showing choanocytes (cho) trapping large bacterial cells (white arrows) which are being engulfed within vesicles. Note smaller bacterial cells with rod shape lying within the mesohyl (black arrows) with a different morphology than those engulfed by the choanocytes

Given the differences in *Ca*. H. symbioticus load across reproductive conditions in May, tissues from non-reproductive individuals, males, and females with late oocytes or early embryos from that month were examined for signs of symbiont phagocytosis. In females with late oocytes and embryos, nurse cells near the periphery of the oocytes and embryos phagocytosed bacteria from the surrounding extracellular collagenous matrix and digested them, contributing to yolk formation, which could be subsequently transferred to the developing oocyte (Fig. 6A–E). Nurse cells are transdifferentiated archeocytes or choanocytes responsible for oocyte provision [30, 88], with a similar morphology to archaeocytes but without a nucleolus in the nucleus (Fig. 6B–C). In females with early embryos, accumulations of small rod-shaped bacteria were observed in the vicinity of the developing embryos, and nurse cells phagocytosed those groups of the accumulated bacteria (Fig. 6A–C). These phagocytosed rod-shaped bacteria were small (less than 1 μm), rod-shaped cells, containing very small clear inclusions or granules, with an appearance similar to other known Rhodobacterales [89, 90]. Putative symbiotic bacteria were frequently observed in the mesohyl of the sponge across stages (Fig. 6A, C, F), and were very different from the trapped bacteria observed within the choanocytes, which were larger, cyanobacteria-like cells (Fig. 6F). Instances of phagocytosis of small Rhodobacterales-like bacteria within the mesohyl were not observed in non-reproductive individuals or males during spermatogenesis.

### Co-expression patterns of the host sponge

**Fig. 7:**
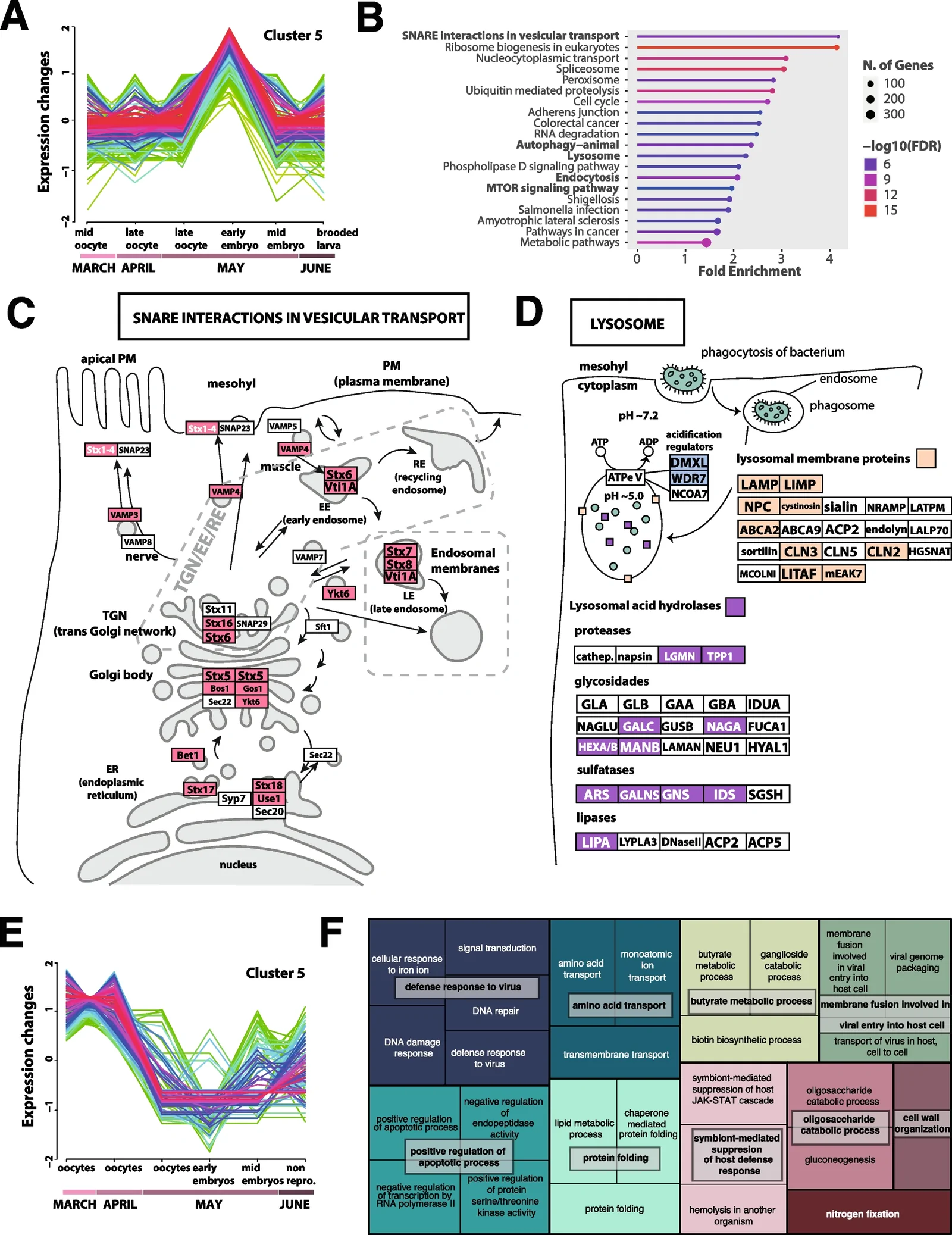
Host (**A**–**D**) and *Ca*. H. symbioticus (**E**–**F**) co-expression patterns during embryogenesis. **A** Cluster of co-expressed sponge genes during embryogenesis in females. Red shades indicate clusters with higher membership (clusters with a large core of tightly co-regulated genes) and green shades with lower values of membership. **B** KEGG pathways enriched within the genes contained in the sponge cluster 5. Pathways related to phagocytosis are indicated in bold. **C **Genes involved within the SNARE interactions in vesicular transport pathways. Colored genes are found within cluster 5. **D** Genes involved within the Lysosome pathway. Colored genes are found within cluster 5. **E** Cluster of co-expressed genes downregulated during embryogenesis in the *Ca*. H. symbioticus community for females. The figure shows the cluster expression changes in *Ca*. H. symbioticus throughout the reproductive period. Red shades indicate clusters with higher membership (clusters with a large core of tightly co-regulated genes) and green shades with lower values of membership. **F** KEGG pathways contained within *Ca*. H. symbioticus cluster 5

We then examined the gene expression profile of individuals during early embryogenesis to identify potential genes responsible for influencing the bacterial symbiotic population and to further support the ultrastructural observations of bacterial phagocytosis during this stage. Clusters of co-regulated genes were subsequently analyzed across the female reproductive cycle for the sponge host, encompassing all available expression stages: mid-oocytes, late oocytes, early embryos, mid-embryos, and brooded larvae. An optimal number of seven clusters was identified by *Mfuzz* (Supplementary Fig. S4, Supplementary Table S8). Among those clusters, cluster 5 contained 7,894 genes specifically increasing their expression in early embryos (Fig. 7A), and 2150 of them were specific to this cluster (Supplementary Fig. S4A). These genes were enriched in several KEGG pathways, including lysosome and SNAREs vesicular transport, involved in both phagocytosis and lysis of bacterial cells, with 50% and 75% of the genes, respectively, belonging to these co-upregulated pathways in cluster 5 (Fig. 7B–D). The category of enrichment related to SNARE vesicular transport was unique for this cluster, with the genes *BET1, STX18. STX8, VAMP1,* and *VAMP4* responsible for the enrichment in KEGG (Fig. 7C; Supplementary Fig. S4A, E). We also detected enrichment of mTOR signaling pathway, which might be involved in phagosome digestion (Fig. 7B). Several other genes involved in mounting an immune response and recognition of microbial peptides were also part of cluster 5, including *MyD88, DMBT1, LMAP2* (Supplementary Table S8). This gene expression profile in the early embryo stage is consistent with the ultrastructural observations of bacterial phagocytosis during early embryogenesis.

### Co-expression patterns of *Ca*. H. symbioticus

Clusters of co-expressed genes for the main symbiont were analyzed using *Mfuzz*, focusing on those displaying similar expression patterns in females producing oocytes, early embryos, and mid embryos during May (Supplementary Fig. S5). An optimal number of seven clusters was identified by *Mfuzz* (Supplementary Fig. S5, Supplementary Table S9). Among them, cluster 5 was characterized by high gene expression in March and April, followed by a marked downregulation in May, particularly in females containing early embryos (Fig. 6E, Supplementary Fig. S5). We combined the annotation from the *Ca.* H. symbioticus MAG (Supplementary Fig. S6) with that obtained from FANTASIA and retrieved the GO terms for the 317 genes contained in cluster 5. This cluster was enriched in biological processes related to DNA repair, nitrogen fixation, protein folding, and lipid metabolism among others; but more importantly, the categories cell wall organization, symbiont-mediated suppression of host defense response, and membrane fusion involved in viral entry into host cell were co-downregulated in the females in May for *Ca.* H. symbioticus (Fig. 6F, Supplementary Table S9). In addition, GO enrichments based on the gene assignments using IMG showed also a response to antibiotics and several of the co-downregulated transcripts were related to porins (Supplementary Table S9).

## Discussion

### Evidence of symbiont phagocytosis during early embryogenesis: from symbiont to food source

**Fig. 8:**
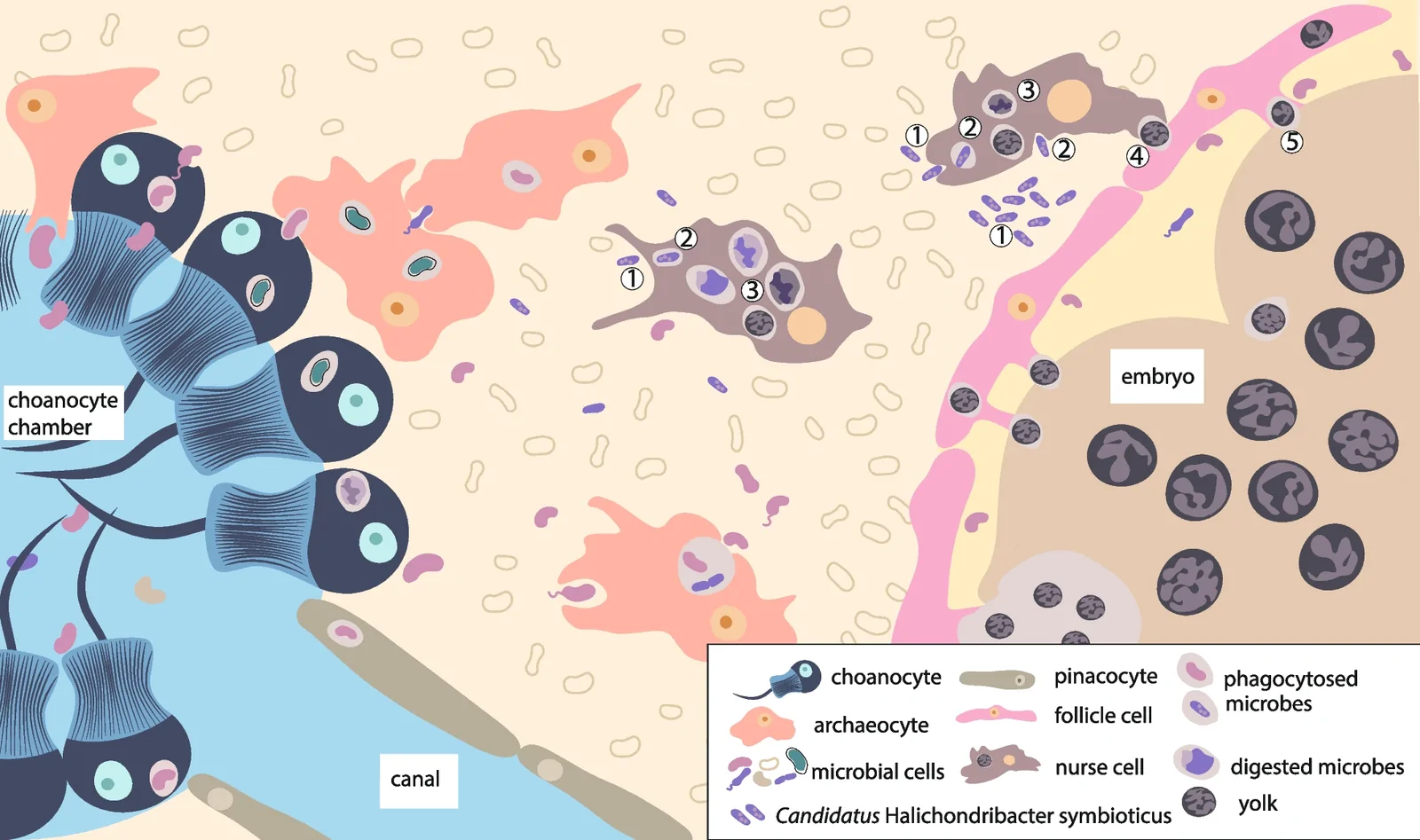
Schematic drawing for the hypothesis for symbiotic bacterial uptake in the mesohyl by nurse cells and digestion for yolk production. **1** Symbiont (*Ca* H. symbioticus) inhabiting the mesohyl of *H. panicea*. **2** Nurse cell phagocytosing symbionts from the mesohyl. **3** Yolk formation from symbiont digestion inside a vesicle of the nurse cell. **4** Yolk being transferred from nurse cell to embryo. Figure adapted from Diez-Vives et al. (2022) [30]

Our findings reveal phagocytosis of the sponge microbial community during embryogenesis, indicating regulation of *Ca.* H. symbioticus by *H. panicea* and its potential role as a nutritional substitute during sponge development (Fig. 8). Likewise, phagocytosis of the main symbiont in the sponge *A. queenslandica* has been observed from the very early stages of the embryo development and throughout the remaining life cycle [32]. In the case of *A. queenslandica,* the symbiotic bacteria phagocytized by the sponge larvae are responsible for the arginine biosynthesis required for larvae settlement and metamorphosis, emphasizing the important role of microbes during development [31]. While *Ca.* H. symbioticus dominated the microbial community of *H. panicea* throughout the reproduction period, its total abundance varied over time, with developmental stage-specific differences detected only in May, when the first steps of embryogenesis occurred (Fig. 3A). A quantitative assessment of symbiont abundance revealed a significant decrease in *Ca*. H. symbioticus during early embryogenesis, as individuals with late oocytes and early embryos harbored fewer copies of this main symbiont compared to male individuals (Fig. 4A). However, total bacterial levels remained stable during early embryogenesis (May), suggesting intracellular processing of *Ca.* H. symbioticus.

Direct ultrastructural evidence further supports this hypothesis, as in females, nurse cells in close vicinity of early embryos were observed phagocytosing bacterial aggregations. Although we cannot unambiguously say that the aggregations of bacteria near the developing embryos were *Ca.* H. symbioticus, the morphology of the cells was highly similar to other Rhodobacterales observed by TEM [89–91]. Importantly, if these microbes were being incorporated into the embryos exclusively as a means of vertical transmission, they would still be detectable in our qPCR assays, as vertically transmitted symbionts remain intact within host tissues. Instead, we observed a specific decline in symbiont abundance across reproductive stages, consistent with microbial digestion, as observed in TEM images (Fig. 6B–E). This pattern strongly suggests that at least part of the symbiont population is processed intracellularly and may contribute to nutritional resources during embryogenesis. Instances of symbiont digestion to form yolk have been reported in the agelasid sponge *Cymbaxinella damicornis* ([92] Figs. 3 and 4) and in the calcarean *Paraleucilla magna* ([93] Fig. 3). Moreover, symbiont digestion during reproduction has been described for two other sponge species [41, 94]. While Oren et al. (2005) [94] suggested that symbiont digestion might be a balancing mechanism of symbiont population size, Díez-Vives et al. (2022) [30] already suggested that this could be a mechanism to provide extra nutrition during embryogenesis. Indeed, symbiont digestion and phagocytosis are common strategies by which host cells acquire nutrients from their partners and it has been widely described in eukaryotes [95, 96]. In some cases, symbiont digestion involves a prior step of symbiont aggregation actively promoted by the host, who is then cultivating its microbial partners to secure a renewable source of nutrients and other resources [95]. This kind of interaction is known as “farming symbiosis”, in which, despite partial digestion of the symbiont population, enough cells persist to maintain the association, ensuring long-term co-evolution between host and symbiont [95]. Many examples of farming symbiosis are found among fungus-farming insects [97–100], though bacterial farming has also been documented. For instance, *Dictyostelium* amoebae establish a stable farming symbiosis with *Burkholderia* bacteria under food-limiting conditions [101]. Even the origin of mitochondria has been linked to a farming hypothesis, wherein the mitochondrial ancestor was initially captured by a phagocytic host as a nutrient source during periods of prey scarcity [102]. Although active farming of *H. panicea* symbionts might not be occurring, it seems likely that *Ca.* H. symbioticus is being processed as a potential nutritional resource during high energetically demanding periods. A similar mechanism has been reported in deep-sea mussels, where symbiont digestion via phagosomes occurs when nutrient availability from bacterial partners declines [103]. Likewise, shifts in microbial symbiont relative abundance during echinoid larval development are understood as related to coping with reduced exogenous resources, suggesting that larvae without a formed digestive system can use their associated microbes for survival [15].

While these cases suggest that nutrient limitation can trigger symbiont consumption, the biological mechanisms regulating this process remain poorly understood. Insights from insects suggest that embryogenesis itself may be a key driver [104, 105]. In these species, massive symbiont aggregations, known as mycetomes, form during embryogenesis, facilitating symbiont digestion and nutrient release to form yolk for the developing embryo [105]. In sponges, symbiont phagocytosis is well documented [41, 88, 94, 106, 107] and an increase in phagocytic activity has been observed in reproductive individuals [36, 88] and in early stages of embryogenesis [32]. Although it has been suggested that phagocytosis might be a selective process, targeting specific symbionts during vitellogenesis in female sponges (e.g., [36]), direct evidence remains scarce. In this study, we provide new evidence that in *H. panicea*, the high nutritional demands during early embryogenesis might trigger the consumption of its microbial community.

### Genomic evidence of symbiont phagocytosis

Phagocytosis is a fundamental component of the innate immune system, responsible for the recognition, ingestion, and elimination of foreign particles, including microorganisms and apoptotic cells [108–110]. This process involves several distinct phases, including particle detection, activation of internalization, and phagosome formation and maturation [108]. During these stages, specific genes are activated to detect target particles and initiate an immune response [110]. Central to this process is the immune system’s capacity to discriminate between self and non-self-entities, a function mediated by pattern recognition receptors (PRRs) that recognize conserved microbial signatures, or pathogen-associated molecular patterns (PAMPs), to initiate a response [111–113].

Our findings suggest that this initial phase of phagocytosis might be occurring during early embryogenesis in *H. panicea*. Specifically, GO terms related to “detection of bacterium” and “immune system development” were significantly upregulated in females during May, coinciding with the beginning of embryogenesis (Fig. 2B). Further support comes from the high expression of specific PRRs, including lectins (*LECA*) and *LBP*, which were exclusively expressed in females during this period (Supplementary Table S1). This concurrent upregulation of lectins and *LBP*, along with ultrastructural observations of bacteria agglutinated near developing embryos (Fig. 6A), indicates that mechanisms of microbial recognition and aggregation are active during this period, potentially priming bacteria for phagocytosis.

Lectins, in particular, are well-known mediators of symbiont recognition and innate immunity. In plants, they enhance macrophage-like phagocytic activity during microbial infection [114, 115]. In animals, lectins have been implicated in microbial defense mechanisms across animals (e.g., fish [116], mussels [117], corals [118], shrimps [119], and nematodes [120]). Additionally, lectins are known for their strong agglutination properties, as seen in shrimp lectins that selectively agglutinate pathogenic *Vibrio* species [119] and in lepidopterans, where a specific lectin agglutinates a broad range of bacterial species [121]. In sponges, lectins are thought to play a role in host defense and potentially in mediating host-microbe interactions [122].

In addition to lectins, we also detected upregulation of the PRR *LECA*, which can function agglutinating cells, a basic immune-like mechanism occurring in females during embryonic development. Also, *LBP* was upregulated, and this receptor binds LPS from Gram-negative bacteria, forming a complex and transferring it to macrophages [123, 124].

Further evidence supporting a role for immune-regulated phagocytosis during development comes from other sponge studies. In *A. queenslandica*, immune receptors are upregulated from embryonic to larval stages, coinciding with phagocytosis of a key symbiont involved in larval settlement [31, 32]. Moreover, the exposure of this sponge to native symbiotic bacteria elicits a rapid immune response consistent with phagocytic activity, whereas exposure to non-native bacteria does not trigger immune gene upregulation [112]. Similarly, the exposure to microbial-associated molecular patterns (MAMPs) induced a species-specific immune response in *Dysidea avara* and *Aplysina aerophoba*, coupled with the stimulation of phagocytosis in the latter one [125, 126]. Moreover, Schmittmann et al. (2021) [72] reported constitutive expression of immune genes upon LPS exposure, but with pronounced individual-specific variability in gene expression profiles. Additionally, evidence of phagocytosis upon particle exposure was empirically demonstrated for our model sponge [127]. According to our results, we suggest that differences in the immune repertoire might be driven by the host biological state, especially during reproductive periods.

In our dataset, we also observed enrichment of GO terms related to “positive regulation of pinocytosis” in females during embryogenesis (Fig. 2B). Following microbial recognition, endocytosis, macropinocytosis, phagocytosis, and autophagy can mediate internalization [110]. Pinocytosis, often referred to as “cell drinking,” allows cells to internalize extracellular fluid and nutrients, subsequently processing them via the endocytic pathway [110, 128]. Notably, a cluster of co-expressed genes in the host (Cluster 5) exhibited high expression in female individuals during embryogenesis, with enrichment for pathways involved in internalization processes, including SNARE interactions, autophagy, lysosome formation, endocytosis, and mTORC1 signaling (Fig. 7B). These mechanisms collectively contribute to the engulfment and degradation of cytosolic material or microorganisms, ultimately facilitating nutrient acquisition [110]. Among these, SNARE-mediated vesicular transport exhibited the highest enrichment. In the endocytic pathway, SNARE proteins regulate intracellular trafficking between endosomes, phagosomes, lysosomes, the Golgi apparatus, the plasma membrane, and the endoplasmic reticulum. SNARE complexity has been extensively studied in arbuscular mycorrhizal symbiosis, revealing differences in the disassembly of symbiotic versus non-symbiotic SNARE complexes [129, 130]. Similarly, in legume-rhizobia interactions, distinct isoforms of t-SNAREs play divergent roles: one isoform is essential for the maturation of symbiosomes into functional nitrogen-fixing forms, while another is involved in cellular processes unrelated to symbiosis [131].

Another key finding in our study is the enrichment of the mechanistic target of rapamycin complex 1 (mTORC1), a central regulator of cellular growth and metabolism that functions via lysosomal nutrient sensing [132, 133]. A compelling example of mTORC1’s role in symbiont digestion is observed in the deep-sea mussel *Bathymodiolus japonicus*, where mTORC1 promotes phagosomal digestion of symbionts under reduced nutrient supply from the symbiont [103]. Tame et al. (2023) [103] demonstrated that when mussels were subjected to methane-depleted environments, they transitioned from relying on symbiont-secreted nutrients to directly digesting symbionts for sustenance. These findings suggest that mTORC1-mediated nutrient signaling plays a crucial role in regulating intracellular symbiosis in deep-sea mussels.

In line with this, we detected significant overexpression of the V-type proton ATPase (*VATL*) in *H. panicea* females during embryogenesis. mTORC1 plays a crucial role in sensing amino acid availability, requiring translocation to the lysosomal surface to become activated [132, 133]. Zoncu et al. (2011) [133] demonstrated that V-ATPase is essential for amino acid-induced activation of mTORC1, identifying it as a key component of the mTOR pathway and highlighting a lysosome-associated machinery for amino acid sensing. This raises the possibility that V-ATPase upregulation during embryogenesis in *H. panicea* may facilitate mTORC1 activation, potentially linking symbiont phagocytosis to nutrient sensing and metabolic regulation.

Moreover, V-ATPase is a key driver of phagosomal acidification [109, 134], a critical step for activating lysosomal hydrolases that mediate bacterial digestion within phagosomes [109]. Acidified phagosomes containing engulfed bacteria have been documented in deep-sea mussels and vesicomyid clams [135, 136]. In *Bathymodiolus japonicus*, symbiosomes containing resident symbionts remained non-acidified, whereas vacuoles containing foreign bacteria exhibited acidification [137]. Consistent with these findings, our transcriptomic analyses revealed a coordinated response between *H. panicea* and its microbial symbionts, with symbiont gene expression in females containing early embryos being significantly enriched for responses to acidic pH, aligning with the acidification necessary for phagocytosis. Further support comes from our *Mfuzz* clustering analysis, which showed co-downregulation of symbiont genes involved in the suppression of host defense responses and membrane fusion (Fig. 7). This pattern suggests that symbionts may actively reduce their immunogenic profile, thereby facilitating host-mediated phagocytosis. Additionally, transcripts associated with porins (outer membrane proteins implicated in bacterial adhesion, invasion, intracellular persistence, and immune evasion [138]) were also downregulated. Given their role in modulating host-microbe interactions, the decreased expression of porin-related genes may contribute to reduced bacterial resistance to host phagocytic mechanisms, thus promoting symbiont uptake and degradation. In sponges, bacterial Ankyrin-repeat proteins (ARPs) have been shown to interfere with host phagocytosis and are thought to serve as a mechanism for evading digestion [139]. In *A. queenslandica*, numerous ARPs were upregulated during the developmental transition from free-swimming larvae to post-larvae, when symbiont restructuring occurs [32]. While we did not observe specific GO enrichment for ARPs in our dataset, these genes were present in *Ca.* H. symbioticus Cluster 5, which was broadly downregulated in females during early embryogenesis in May. This downregulation may similarly reflect a loss of anti-phagocytic capabilities in symbionts, thereby facilitating their elimination by the host. It is worth acknowledging that embryogenesis is inherently associated with extensive cellular remodeling, including autophagy, yolk processing, and lysosome-mediated turnover of maternal components. Therefore, the host transcriptomic signatures could partially reflect general developmental processes rather than being exclusively attributable to symbiont digestion. However, the microbial metatranscriptomic data explained above provide a line of evidence that is independent of host developmental state and cannot be explained by embryogenic remodeling alone. Future studies employing enzyme cytochemical approaches (e.g., esterase or phosphatase activity staining) combined with immunolocalization of candidate digestive proteins identified here would provide valuable spatial validation of the phagocytic digestion model proposed, and represent a natural next step for this system.

## Conclusions

Our study provides evidence that *H. panicea* potentially engages in phagocytosis of its microbial community, during early embryogenesis. The diminished number of co-expressed genes within the *Ca.* H. symbioticus cluster during this period suggests a breakdown in metabolic interdependence between the host and its microbial community. This supports the hypothesis that, during this energetically demanding developmental stage, *H. panicea* shifts its nutritional strategy from relying on symbiont-secreted metabolites to processing symbionts as a potential nutritional resource. Both transcriptomic and ultrastructural data converge to support a regulated, immune-mediated phagocytic pathway, marked by the upregulation of pattern recognition receptors, vesicular trafficking machinery, lysosomal acidification, and nutrient-sensing pathways. Simultaneously, the downregulation of key symbiont genes suggests a reduced capacity to resist host immunity, thereby facilitating their degradation. Thus, we suggest that *H. panicea* microbial partners might serve as mutualist symbionts that might provide nutritional reserves during early embryogenesis. By highlighting this function, our study advances the understanding of host-microbe dynamics during animal development and uncovers a previously underappreciated role that may involve intracellular symbiont digestion in supporting early embryogenesis in basal metazoans.

## Supplementary Information

Supplementary Material 1: Supplementary Figure S1. Rarefaction curves of microbiome data for all the study samples. Dashed line indicates the minimum reads threshold used for rarefaction.

Supplementary Material 2: Supplementary Figure S2. Percentage of *H.panicea *population involved in reproduction throughout the reproductive period. Percentage of females, males or non-reproductive individuals is shown in different colors for each month.

Supplementary Material 3: Supplementary Figure S3. *H. panicea *microbiome A) Barplots showing the top 15 taxa at class level for each sample across months. Abundance of the dominant *Ca*. H. symbioticus is shown as a separate entity. All the other taxa are grouped as “Others”. B) PCoA of all the samples colored according to sampling month. Shape indicates individual sex (Female, Male or Non-reproductive). Variation explained by the first two axes is indicated as %. C) Boxplot showing the Shannon diversity for each month separated by reproductive state. D) Shannon diversity across months. E) Richness across months.

Supplementary Material 4: Supplementary Figure S4. Sponge gene clusters obtained with Mfuzz. A) Number of shared genes between the different clusters. B) Clusters of the host co-expressed genes along the reproductive period (B–H).

Supplementary Material 5: Supplementary Figure S5.*Ca.*H. symbioticus gene clusters obtained with Mfuzz.

Supplementary Material 6: Supplementary Figure S6. Metagenome of *Ca*. H. symbioticus. A Circos plot for the metagenome of *Ca*. H. symbioticus with summary statistics for regions that were or were not coding regions (CDS), were tRNA and rRNA genes, guanine (G) and cytosine (C) content and skew, and Cluster of Orthologous Groups (COGs) cellular processes and signaling ([D] Cell cycle control, cell division, chromosome partitioning, [M] Cell wall/membrane/envelope biogenesis, [N] Cell motility, [O] Posttranslational modification, protein turnover, chaperones, [T] Signal transduction mechanisms, [U] Intracellular trafficking, secretion, and vesicular transport, [V] Defense mechanisms, and [Z] Cytoskeleton), information storage and processing ([B] Chromatin structure and dynamics, [J] Translation, ribosomal structure and biogenesis, [K] Transcription, and [L] Replication, recombination and repair), metabolism ([C] Energy production and conversion, [E] Amino acid transport and metabolism, [F] Nucleotide transport and metabolism, [G] Carbohydrate transport and metabolism, [H] Coenzyme transport and metabolism, [I] Lipid transport and metabolism, [P] Inorganic ion transport and metabolism, and [Q] Secondary metabolites biosynthesis, transport and catabolism), and those that were poorly characterized ([R] General function prediction only and [S] Function unknown).

Supplementary Material 7: Supplementary Table S1. Differentially Expressed (DE) transcripts in *H. panicea.* Tables of the DE host transcripts for each month (separated spreadsheets). In each table, values for LogFC, p-value, FDR and Expression values for each transcript are shown. Gene annotations and descriptions are shown in the last columns.

Supplementary Material 8: Supplementary Table S2. Gene Ontologies (GO) enrichments of host transcriptome. Tables showing the GO terms enriched for females or males for each month (separated spreadsheets).

Supplementary Material 9: Supplementary Table S3. *H. panicea *microbiome data. A) Taxonomic composition at Phylum level for each month and reproductive condition ordered in descending mean total abundance (last column). Taxa with relative abundances < 0.05 are grouped as “Others”. B) Taxonomic composition at Class level for each month and reproductive condition ordered in descending mean total abundance (last column). Taxa with relative abundances < 0.05 are grouped as “Others”. C) Top 15 taxa at Class level for each month and reproductive condition ordered in descending mean total abundance (last column). Abundance of the dominant *Ca.* H. symbioticus is shown as a separate entity. All the other taxa are grouped as “Others”. D) Alpha diversity measures (Richness, Shannon and Inverse Simpson) for each sample. An information table for each individual is provided. E) Number of Differentially Abundant (DA) ZOTUs between reproductive and non-reproductive individuals for each month.

Supplementary Material 10: Supplementary Table S4. Transcriptome Statistics. A) General transcriptome descriptors. B) Number of raw, filtered and mapped reads for each sample. C) Expression counts table for Metazoan transcripts. D) Expression counts table for microbial transcripts.

Supplementary Material 11: Supplementary Table S5. Differentially Expressed (DE) transcripts of *H. panicea *microbiome. Tables of the DE host transcripts for each month (separated spreadsheets). In each table, values for LogFC, p-value, FDR and Expression values for each transcript are shown. Gene annotations and descriptions are shown in the last columns.

Supplementary Material 12: Supplementary Table S6. Gene Ontologies (GO) enrichments of microbial transcriptomes. Tables showing the GO terms enriched for females or males for each month (separated spreadsheets).

Supplementary Material 13: Supplementary Table S7.qPCRstatistical results. Quantitative estimates of the abundances of total bacteria and *Ca*. H. symbioticus obtained with qPCR. Results of the statistical analyses comparing reproductive conditions in each month are shown.

Supplementary Material 14: Supplementary Table S8. Mfuzz host clusters. Tables showing the number of genes contained within each cluster. The transcripts and their gene annotations are shown in separate spreadsheets for each cluster (C1–C7).

Supplementary Material 15: Supplementary Table S9. Mfuzz Cluster 5 Transcripts, Gene Annotations and Corresponding Modules of Co-expressed Transcripts Found in Cluster 5 for *Ca*. H. symbioticus.

Supplementary Material 16: Supplementary Table S10. Information table of the sample reproductive state and its correspondence with Amplicon data (spreadsheet 1) and Metatranscriptomic data (spreadsheet 2).

Supplementary Material 17: Supplementary Statistics. Statistics for 16S rRNA gene amplicon data for beta and alpha diversity analyses.

## Data Availability

Amplicon sequencing and metatranscriptomic data generated in this study are available in the NCBI Sequence Read Archive under BioProject accession PRJNA1274490. Correspondence between samples and datasets is provided in Supplementary Table 10. Quantitative PCR (qPCR) data are available in Supplementary Table 7. All scripts used for data processing and analysis are publicly available in a GitHub repository (https://github.com/martaturon/hpanicea-microbiome).
