# Supplementary material for: Phagocytosis of microbial symbionts supports embryonic nutrition in the sponge Halichondria panicea
Source: Microbiome. 2026 Aug 26;14:211. doi: 10.1186/s40168-026-02473-y (PMC13543439; doi:10.1186/s40168-026-02473-y)
Supplement: Supplementary file 1 [file 40168_2026_2473_MOESM1_ESM.pdf]

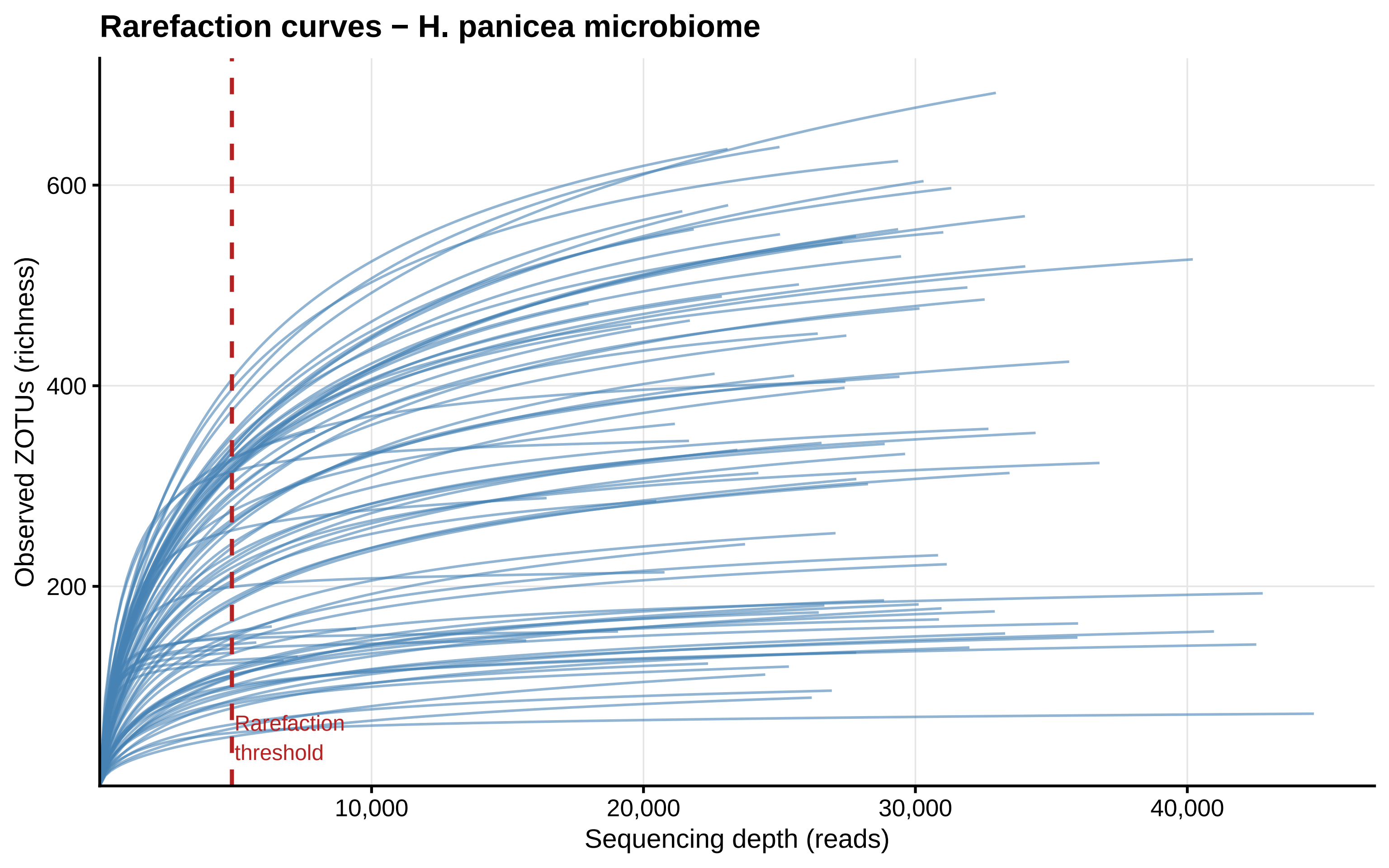

Rarefaction curves − H. panicea microbiome
Observed ZOTUs (richness)
600
400
200
Rarefaction
threshold
10,000
20,000
30,000
40,000
Sequencing depth (reads)
