# Supplementary material for: Phagocytosis of microbial symbionts supports embryonic nutrition in the sponge Halichondria panicea
Source: Microbiome. 2026 Aug 26;14:211. doi: 10.1186/s40168-026-02473-y (PMC13543439; doi:10.1186/s40168-026-02473-y)
Supplement: Supplementary file 2 [file 40168_2026_2473_MOESM2_ESM.pdf]

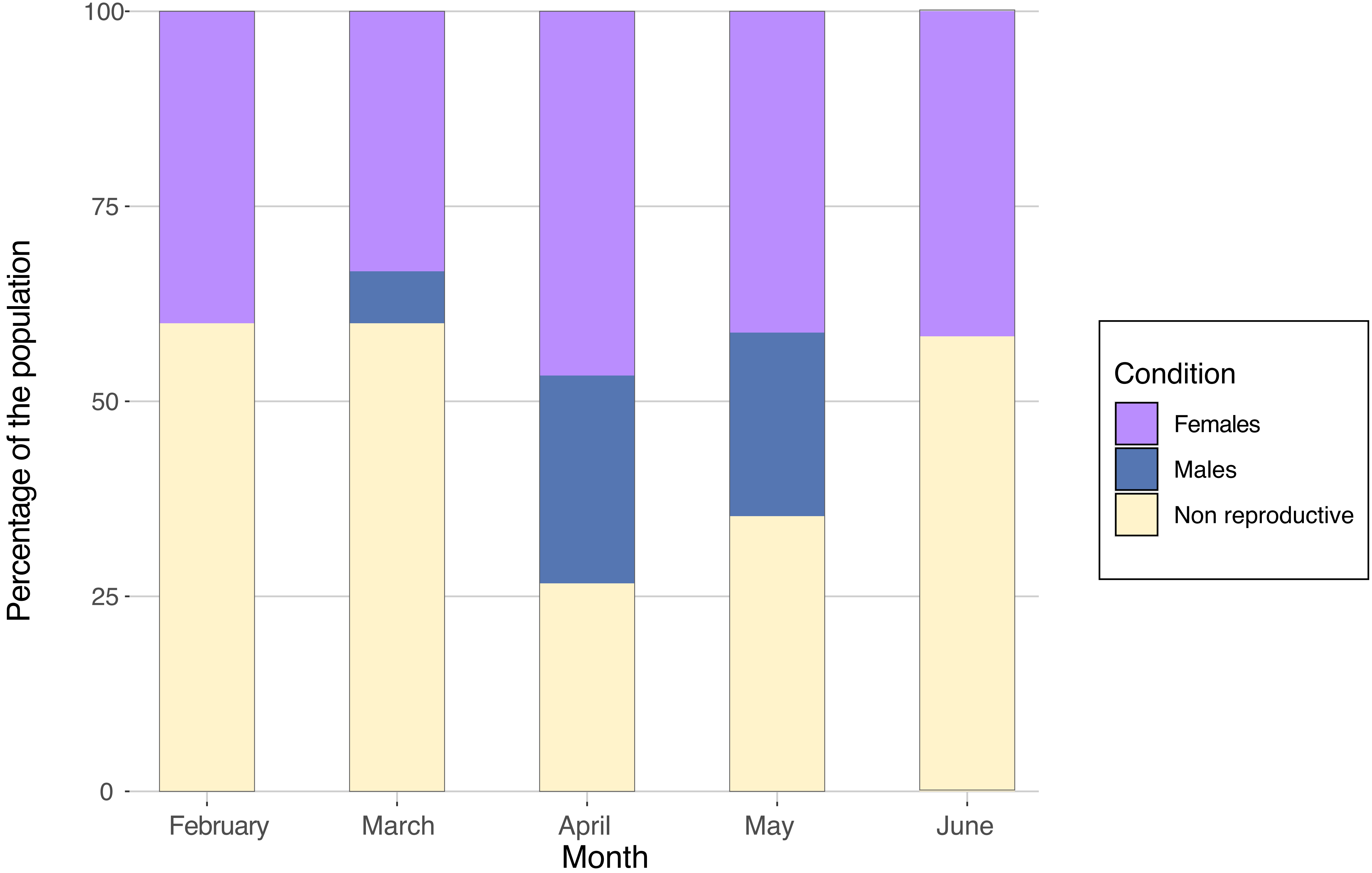

Percentage of the population
100
75
50
25
0
February
March
April
May
June
Month
Condition
Females
Males
Non reproductive
