# Supplementary material for: Phagocytosis of microbial symbionts supports embryonic nutrition in the sponge Halichondria panicea
Source: Microbiome. 2026 Aug 26;14:211. doi: 10.1186/s40168-026-02473-y (PMC13543439; doi:10.1186/s40168-026-02473-y)
Supplement: Supplementary file 3 [file 40168_2026_2473_MOESM3_ESM.pdf]

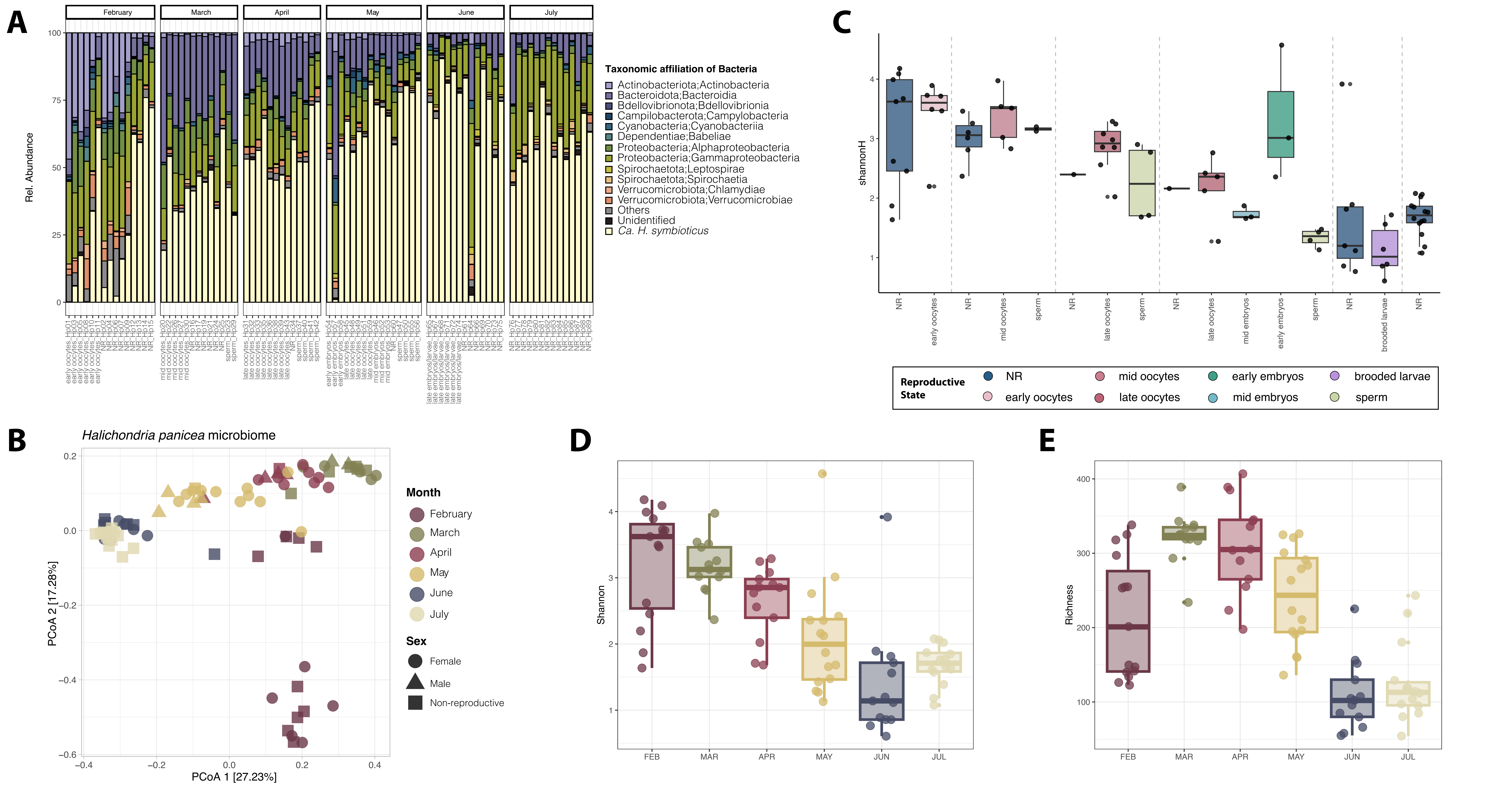
A
February
March
April
May
June
July
Rel. Abundance
Taxonomic affiliation of Bacteria
Actinobacteriota;Actinobacteria
Bacteroidota;Bacteroidia
Bdellovibrionota;Bdellovibrionia
Campilobacterota;Campylobacteria
Cyanobacteria;Cyanobacteriia
Dependentiae;Babeliae
Proteobacteria;Alphaproteobacteria
Proteobacteria;Gammaproteobacteria
Spirochaetota;Leptospirae
Spirochaetota;Spirochaetia
Verrucomicrobiota;Chlamydiae
Verrucomicrobiota;Verrucomicrobiae
Others
Unidentified
Ca. H. symbioticus
C
shannonH
NR
early oocytes
mid oocytes
sperm
late oocytes
mid embryos
early embryos
brooded larvae
Reproductive State
B
Halichondria panicea microbiome
PCoA 1 [27.23%]
PCoA 2 [17.28%]
Month
February
March
April
May
June
July
Sex
Female
Male
Non-reproductive
D
Shannon
FEB
MAR
APR
MAY
JUN
JUL
E
Richness
FEB
MAR
APR
MAY
JUN
JUL
