# Supplementary material for: Phagocytosis of microbial symbionts supports embryonic nutrition in the sponge Halichondria panicea
Source: Microbiome. 2026 Aug 26;14:211. doi: 10.1186/s40168-026-02473-y (PMC13543439; doi:10.1186/s40168-026-02473-y)
Supplement: Supplementary file 4 [file 40168_2026_2473_MOESM4_ESM.pdf]

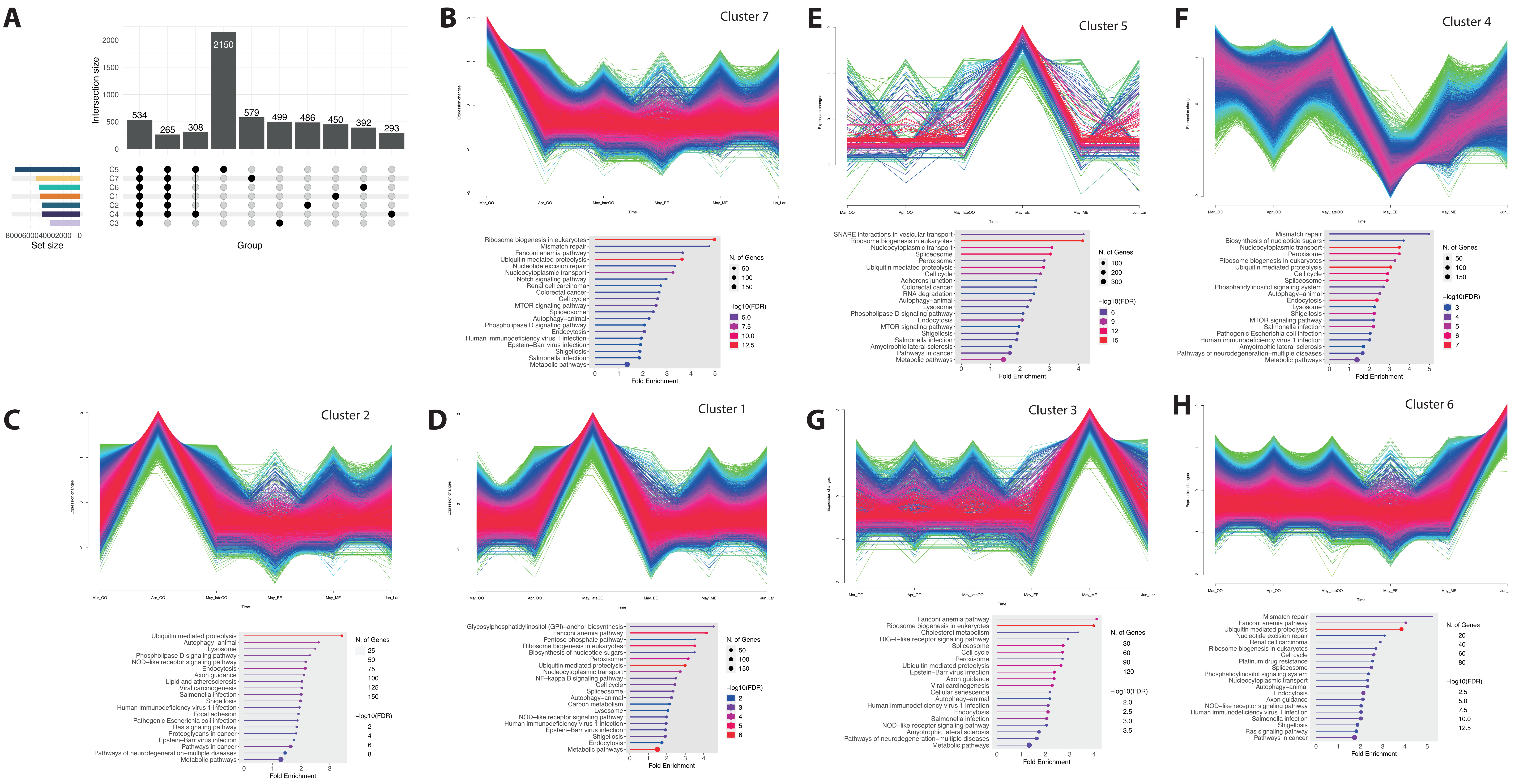
A
Intersection size
2150
534
265
308
579
499
486
450
392
293
C5
C7
C6
C1
C2
C4
C3
Set size
Group
B
Cluster 7
Expression changes
Time
Mar_OO
Apr_OO
May_lateOO
May_EE
May_ME
Jun_Lar
Ribosome biogenesis in eukaryotes
Mismatch repair
Fanconi anemia pathway
Ubiquitin mediated proteolysis
Nucleotide excision repair
Nucleocytoplasmic transport
Notch signaling pathway
Renal cell carcinoma
Colorectal cancer
Cell cycle
MTOR signaling pathway
Spliceosome
Autophagy−animal
Phospholipase D signaling pathway
Endocytosis
Human immunodeficiency virus 1 infection
Epstein−Barr virus infection
Shigellosis
Salmonella infection
Metabolic pathways
Fold Enrichment
N. of Genes
−log10(FDR)
E
Cluster 5
SNARE interactions in vesicular transport
Ribosome biogenesis in eukaryotes
Nucleocytoplasmic transport
Spliceosome
Peroxisome
Ubiquitin mediated proteolysis
Cell cycle
Adherens junction
Colorectal cancer
RNA degradation
Autophagy−animal
Lysosome
Phospholipase D signaling pathway
Endocytosis
MTOR signaling pathway
Shigellosis
Salmonella infection
Amyotrophic lateral sclerosis
Pathways in cancer
Metabolic pathways
F
Cluster 4
Mismatch repair
Biosynthesis of nucleotide sugars
Nucleocytoplasmic transport
Peroxisome
Ribosome biogenesis in eukaryotes
Ubiquitin mediated proteolysis
Cell cycle
Spliceosome
Phosphatidylinositol signaling system
Autophagy−animal
Endocytosis
Lysosome
Shigellosis
MTOR signaling pathway
Salmonella infection
Pathogenic Escherichia coli infection
Human immunodeficiency virus 1 infection
Amyotrophic lateral sclerosis
Pathways of neurodegeneration−multiple diseases
Metabolic pathways
C
Cluster 2
Ubiquitin mediated proteolysis
Autophagy−animal
Lysosome
Phospholipase D signaling pathway
NOD−like receptor signaling pathway
Endocytosis
Axon guidance
Lipid and atherosclerosis
Viral carcinogenesis
Salmonella infection
Shigellosis
Human immunodeficiency virus 1 infection
Focal adhesion
Pathogenic Escherichia coli infection
Ras signaling pathway
Proteoglycans in cancer
Epstein−Barr virus infection
Pathways in cancer
Pathways of neurodegeneration−multiple diseases
Metabolic pathways
D
Cluster 1
Glycosylphosphatidylinositol (GPI)−anchor biosynthesis
Fanconi anemia pathway
Pentose phosphate pathway
Ribosome biogenesis in eukaryotes
Biosynthesis of nucleotide sugars
Peroxisome
Ubiquitin mediated proteolysis
Nucleocytoplasmic transport
NF−kappa B signaling pathway
Cell cycle
Spliceosome
Autophagy−animal
Carbon metabolism
Lysosome
NOD−like receptor signaling pathway
Human immunodeficiency virus 1 infection
Epstein−Barr virus infection
Shigellosis
Endocytosis
Metabolic pathways
G
Cluster 3
Fanconi anemia pathway
Ribosome biogenesis in eukaryotes
Cholesterol metabolism
RIG−I−like receptor signaling pathway
Spliceosome
Cell cycle
Peroxisome
Ubiquitin mediated proteolysis
Epstein−Barr virus infection
Axon guidance
Viral carcinogenesis
Cellular senescence
Autophagy−animal
Human immunodeficiency virus 1 infection
Endocytosis
Salmonella infection
NOD−like receptor signaling pathway
Amyotrophic lateral sclerosis
Pathways of neurodegeneration−multiple diseases
Metabolic pathways
H
Cluster 6
Mismatch repair
Fanconi anemia pathway
Ubiquitin mediated proteolysis
Nucleotide excision repair
Renal cell carcinoma
Ribosome biogenesis in eukaryotes
Cell cycle
Platinum drug resistance
Spliceosome
Phosphatidylinositol signaling system
Nucleocytoplasmic transport
Autophagy−animal
Endocytosis
Axon guidance
NOD−like receptor signaling pathway
Human immunodeficiency virus 1 infection
Salmonella infection
Shigellosis
Ras signaling pathway
Pathways in cancer
