# Supplementary material for: Phagocytosis of microbial symbionts supports embryonic nutrition in the sponge Halichondria panicea
Source: Microbiome. 2026 Aug 26;14:211. doi: 10.1186/s40168-026-02473-y (PMC13543439; doi:10.1186/s40168-026-02473-y)
Supplement: Supplementary file 5 [file 40168_2026_2473_MOESM5_ESM.pdf]

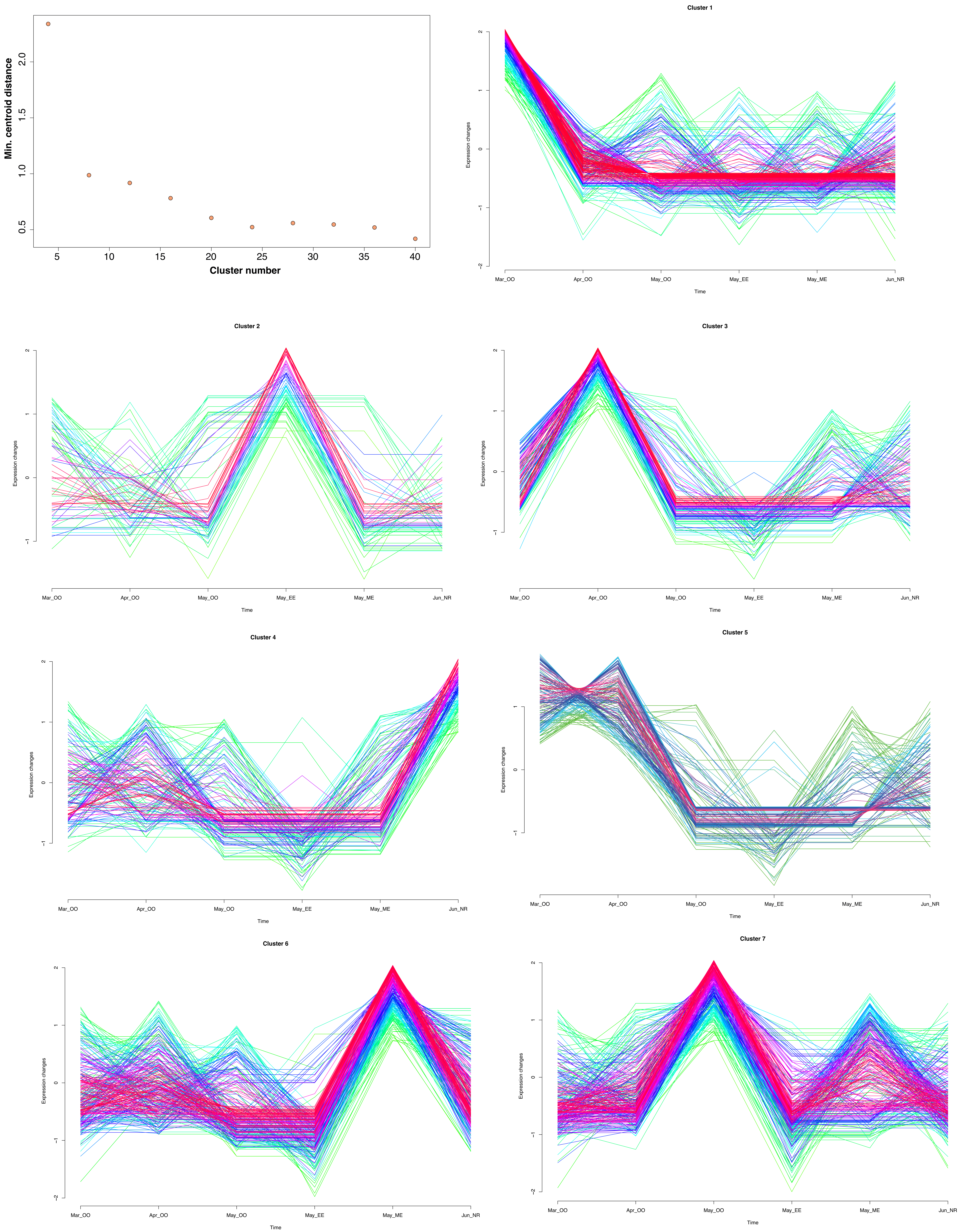
Min. centroid distance
Cluster number
Cluster 1
Cluster 2
Cluster 3
Cluster 4
Cluster 5
Cluster 6
Cluster 7
Expression changes
Time
Mar_OO
Apr_OO
May_OO
May_EE
May_ME
Jun_NR
