# Supplementary material for: Phagocytosis of microbial symbionts supports embryonic nutrition in the sponge Halichondria panicea
Source: Microbiome. 2026 Aug 26;14:211. doi: 10.1186/s40168-026-02473-y (PMC13543439; doi:10.1186/s40168-026-02473-y)
Supplement: Supplementary file 6 [file 40168_2026_2473_MOESM6_ESM.pdf]

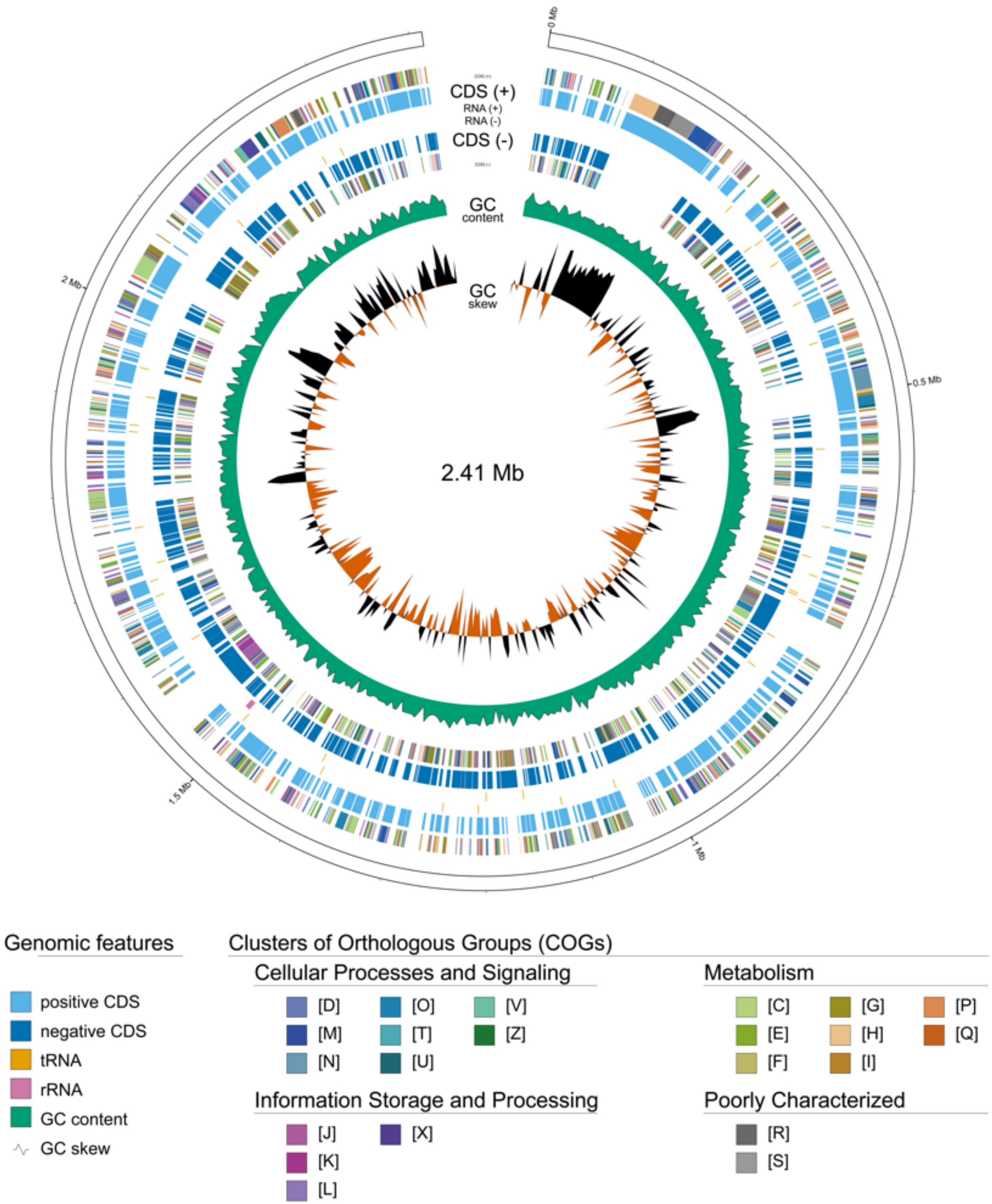
0 Mb
0.5 Mb
1 Mb
1.5 Mb
2 Mb
CDS (+)
RNA (+)
RNA (-)
CDS (-)
GC content
GC skew
2.41 Mb
Genomic features
positive CDS
negative CDS
tRNA
rRNA
GC content
GC skew
Clusters of Orthologous Groups (COGs)
Cellular Processes and Signaling
[D]
[O]
[V]
[M]
[T]
[Z]
[N]
[U]
Metabolism
[C]
[G]
[P]
[E]
[H]
[Q]
[F]
[I]
Information Storage and Processing
[J]
[X]
[K]
[L]
Poorly Characterized
[R]
[S]
