# Supplementary material for: Phagocytosis of microbial symbionts supports embryonic nutrition in the sponge Halichondria panicea
Source: Microbiome. 2026 Aug 26;14:211. doi: 10.1186/s40168-026-02473-y (PMC13543439; doi:10.1186/s40168-026-02473-y)
Supplement: Supplementary file 7 [file 40168_2026_2473_MOESM17_ESM.docx]

**Beta diversity**

***Permanova for date***

Permutation test for adonis under reduced model

Terms added sequentially (first to last)

Permutation: free

Number of permutations: 999

|  | Df | SumOfSqs | R2 | F | Pr(>F) |
| --- | --- | --- | --- | --- | --- |
| Species | 5 | 9.2266 | 0.48928 | 15.137 | **0.001** *** |
| Residual | 79 | 9.6308 | 0.51 |  |  |
| Total | 84 | 18.8574 | 1 |  |  |

---

Signif. codes: 0 ‘***’ 0.001 ‘**’ 0.01 ‘*’ 0.05 ‘.’ 0.1 ‘ ’ 1

***Permanova for date and reproduction (nested factors)***

Permutation test for adonis under reduced model

Terms added sequentially (first to last)

Permutation: free

Number of permutations: 999

adonis2(formula = counts_bc ~ Date * Status2, data = Infotable)

Df SumOfSqs	R2	F Pr(>F)

|  | Df | SumOfSqs | R2 | F | Pr(>F) |
| --- | --- | --- | --- | --- | --- |
| Date | 5 | 9.22 | 0.48 | 15.14 | **0.001** *** |
| State | 1 | 0.12 | 0.006 | 0.98 | 0.354 |
| Date:State | 2 | 0.49 | 0.02 | 1.009 | 0.438 |
| Residual | 74 | 9.01 | 0.47 |  |  |
| Total | 84 | 18.85 | 1 |  |  |

---

Signif. codes: 0 ‘***’ 0.001 ‘**’ 0.01 ‘*’ 0.05 ‘.’ 0.1 ‘ ’ 1

**Dates and reproduction (Betadiversity)**

Summary table showing the results of Permutest and Permanova for each month. Permanova was first performed comparing reproductive vs non-reproductive individuals, and then comparing different reproductive stages (oocytes, sperm, embryos, larvae and non-reproductive).

| **Months** | **n** | **Permutest** | | **Permanova (Repro vs NR)** | | **Permanova**  **(Repro Stage)** | |
| --- | --- | --- | --- | --- | --- | --- | --- |
| Februrary | 15 | F | 0.002 | R^2^ | 0.94 | R^2^ | 0.94 |
|  |  | p-val | 0.951 | p-val | 0.43 | p-val | 0.43 |
| March | 13 | F | 0 | R^2^ | 0.98 | R^2^ | 0.92 |
|  |  | p-val | 0.998 | p-val | 0.41 | p-val | 0.42 |
| April | 14 | F | 0.36 | R^2^ | 0.81 | R^2^ | 1.02 |
|  |  | p-val | 0.58 | p-val | 0.64 | p-val | 0.4 |
| May | 16 | F | 0.05 | R^2^ | 0.82 | R^2^ | 1.14 |
|  |  | p-val | 0.87 | p-val | 0.71 | p-val | 0.2 |
| June |  | F | 0.08 | R^2^ | 1.2 | R^2^ | 1.2 |
|  |  | p-val | 0.85 | p-val | 0.19 | p-val | 0.19 |

**Alpha diversity**

**Shannon:**

**Anova for Date**

|  | Df | SumOfSqs | R2 | F | Pr(>F) |
| --- | --- | --- | --- | --- | --- |
| Date | 5 | 39.48 | 7.89 | 16.6 | **0.001** *** |
| Residual | 79 | 37.46 | 0.47 |  |  |

**Pair-wise comparisons**

Tukey multiple comparisons of means

95% family-wise confidence level

Fit: aov(formula = shannonH ~ Date, data = Infotable)

| Comparison | diff | lwr | upr | p-adj. |
| --- | --- | --- | --- | --- |
| feb-22-abr-22 | 0.636 | -0.112 | 1.383 | 0.141 |
| jul-22-abr-22 | -0.920 | -1.680 | -0.160 | **0.009** |
| jun-22-abr-22 | -1.189 | -1.964 | -0.415 | **0.000** |
| mar-22-abr-22 | 0.551 | -0.224 | 1.326 | 0.310 |
| may-22-abr-22 | -0.473 | -1.209 | 0.263 | 0.423 |
| jul-22-feb-22 | -1.556 | -2.303 | -0.808 | **0.000** |
| jun-22-feb-22 | -1.825 | -2.587 | -1.063 | **0.000** |
| mar-22-feb-22 | -0.085 | -0.847 | 0.678 | 1.000 |
| may-22-feb-22 | -1.109 | -1.832 | -0.386 | **0.000** |
| jun-22-jul-22 | -0.269 | -1.044 | 0.506 | 0.912 |
| mar-22-jul-22 | 1.471 | 0.697 | 2.246 | 0.000 |
| may-22-jul-22 | 0.447 | -0.289 | 1.183 | 0.488 |
| mar-22-jun-22 | 1.740 | 0.951 | 2.529 | **0.000** |
| may-22-jun-22 | 0.716 | -0.035 | 1.467 | 0.070 |
| may-22-mar-22 | -1.024 | -1.775 | -0.273 | **0.002** |

**Alpha diversity each month**

| **Months** | **n** | **Anova Shannon** | | **Anova richness** | |
| --- | --- | --- | --- | --- | --- |
| Februrary | 15 | F | 0.4 | F | 1.02 |
|  |  | p-val | 0.53 | p-val | 0.33 |
| March | 13 | F | 1.22 | F | 1.94 |
|  |  | p-val | 0.33 | p-val | 0.194 |
| April | 13 | F | 2.02 | F | 0.65 |
|  |  | p-val | 0.18 | p-val | 0.54 |
| May | 16 | F | 5.03 | F | 5.75 |
|  |  | p-val | **0.015*** | p-val | **0.009*** |
| June | 13 | F | 1.2 | F | 0.001 |
|  |  | p-val | 0.29 | p-val | 0.97 |

--

Signif. codes: 0 ‘***’ 0.001 ‘**’ 0.01 ‘*’ 0.05 ‘.’ 0.1 ‘ ’ 1

**Pair-wise comparisons Richness May**

Tukey multiple comparisons of means

95% family-wise confidence level

| Comparison | diff | lwr | upr | p.adj |
| --- | --- | --- | --- | --- |
| late oocytes-NR | 6.00 | -145.85 | 157.85 | 1.00 |
| late oocytes/early embryos/mid embryos-NR | -55.67 | -215.73 | 104.40 | 0.79 |
| early embryos-NR | 48.33 | -111.73 | 208.40 | 0.86 |
| sperm-NR | -92.00 | -246.98 | 62.98 | 0.36 |
| late oocytes/early embryos/mid embryos-late oocytes | -61.67 | -162.90 | 39.57 | 0.34 |
| early embryos-late oocytes | 42.33 | -58.90 | 143.57 | 0.67 |
| sperm-late oocytes | -98.00 | -190.99 | -5.01 | **0.04** |
| early embryos-late oocytes/early embryos/mid embryos | 104.00 | -9.18 | 217.18 | 0.08 |
| sperm-late oocytes/early embryos/mid embryos | -36.33 | -142.21 | 69.54 | 0.80 |
| sperm-early embryos | -140.33 | -246.21 | -34.46 | **0.01** |

**Pair-wise comparisons Shannon May**

Tukey multiple comparisons of means

95% family-wise confidence level

|  | diff | lwr | upr | p.adj |
| --- | --- | --- | --- | --- |
| late oocytes-NR | 0.03 | -2.10 | 2.15 | 1.00 |
| late oocytes/early embryos/mid embryos-NR | -0.42 | -2.66 | 1.81 | 0.97 |
| early embryos-NR | 1.15 | -1.08 | 3.39 | 0.49 |
| sperm-NR | -0.83 | -3.00 | 1.34 | 0.73 |
| late oocytes/early embryos/mid embryos-late oocytes | -0.45 | -1.86 | 0.96 | 0.84 |
| early embryos-late oocytes | 1.13 | -0.29 | 2.54 | 0.14 |
| sperm-late oocytes | -0.86 | -2.16 | 0.44 | 0.27 |
| early embryos-late oocytes/early embryos/mid embryos | 1.58 | 0.00 | 3.16 | **0.05** |
| sperm-late oocytes/early embryos/mid embryos | -0.41 | -1.89 | 1.07 | 0.90 |
| sperm-early embryos | -1.98 | -3.46 | -0.50 | **0.01** |
